# Synaptophysin autoantibodies mediate synaptic dysfunction in cerebellar ataxia

**DOI:** 10.1016/j.xcrm.2026.102822

**Published:** 2026-05-18

**Authors:** Samantha Ho, Hoi Kiu Wong, Dorina Shqau, Stephan Winklmeier, Marcus Grobe-Einsler, Kevin Rostasy, Tania Kümpfel, Franziska S. Thaler, Lior Brimberg, Marc A. Schneider, Thomas Muley, Atay Vural, Thomas Seifert-Held, Verena Endmayr, Romana Höftberger, Jennifer Faber, Thomas Klopstock, Silke Frahm, Lisa Ann Gerdes, Edgar Meinl, Simone Mader

**Affiliations:** 1 Institute of Clinical Neuroimmunology, LMU University Hospital, Ludwig-Maximilians-Universität München, 81377 Munich, Germany; 2 Biomedical Center, Medical Faculty, Ludwig-Maximilians-Universität München, 82152 Planegg-Martinsried, Germany; 3 Graduate School of Systemic Neuroscience, Ludwig-Maximilians-Universität München, 82152 Planegg-Martinsried, Germany; 4 Department of Translational Immunology, Department of Medicine 3 – Rheumatology and Immunology, Nikolaus-Fiebiger Center for Molecular Medicine, Friedrich-Alexander-Universität (FAU) Erlangen-Nürnberg, Universitätsklinikum Erlangen, 91054 Erlangen, Germany; 5 German Center for Neurodegenerative Diseases, 53127 Bonn, Germany; 6 Children’s Hospital Datteln, Pediatric Neurology, Witten/Herdecke University, 45711 Datteln, Germany; 7 The Institute for Molecular Medicine, The Feinstein Institutes for Medical Research, Northwell Health, Manhasset, NY 11030, USA; 8 Translational Research Unit, Thoraxklinik at University Hospital Heidelberg, 69126 Heidelberg, Germany; 9 Translational Lung Research Center Heidelberg, German Center for Lung Research (DZL), 69126 Heidelberg, Germany; 10 Koç University Research Center for Translational Medicine (KUTTAM), İstanbul 34450, Türkiye; 11 Koç University Graduate School of Health Sciences, İstanbul 34450, Türkiye; 12 Koç University School of Medicine, Department of Neurology, İstanbul 34450, Türkiye; 13 Department of Neurology, Hospital Murtal, 8720 Knittelfeld, Austria; 14 Department of Neurology, Division of Neuropathology and Neurochemistry, Medical University of Vienna, 1090 Vienna, Austria; 15 Comprehensive Center for Clinical Neurosciences and Mental Health, Medical University of Vienna, 1090 Vienna, Austria; 16 Center of Neurology, Department of Parkinson’s, Sleep and Movement Disorders, University Hospital Bonn, 53127 Bonn, Germany; 17 Department of Neuroradiology, University Hospital Bonn, 53127 Bonn, Germany; 18 Friedrich-Baur-Institute at the Department of Neurology, LMU University Hospital, Ludwig-Maximilians-Universität München, 80336 Munich, Germany; 19 German Center for Neurodegenerative Diseases, 81377 Munich, Germany; 20 Munich Cluster for Systems Neurology (SyNergy), 81377 Munich, Germany; 21 Technology Platform Pluripotent Stem Cells, Max Delbrück Center for Molecular Medicine in the Helmholtz Association, 13125 Berlin, Germany; 22 Deutsches Zentrum Immuntherapie, Friedrich-Alexander-Universität (FAU) Erlangen-Nürnberg and Universitätsklinikum Erlangen, 91054 Erlangen, Germany

**Keywords:** autoantibodies, synaptophysin, cerebellar ataxia, neuroinflammation, autoimmunity, synapse

## Abstract

Cerebellar ataxias (CAs) encompass a wide spectrum of genetic and sporadic origins, of which a portion is driven by immune-mediated pathomechanisms. While some patients harbor known autoantibodies, assisting diagnosis and treatment, many remain seronegative. Here, we identify synaptophysin (SYP) as an autoantigen in CA by serum IgG staining on primate tissue, combined with human protein array and cell-based assay (CBA). SYP is abundant in presynaptic vesicles and is transiently exposed on the cell surface. Out of 43 patients with CA with synaptic serum reactivity on cerebellar sections, SYP antibodies were identified by CBA in 2 patients. Exposure of human induced pluripotent stem cell (iPSC)-derived glutamatergic neurons to patient’s IgG with SYP antibodies causes selective SYP accumulation at the presynaptic membrane. Patient’s IgG and SYP monoclonal antibody reduce neuronal populational activity recorded by multi-electrode array. Altogether, we identify SYP as an autoantigen for further stratifying patients with CA. Our functional experiments with SYP antibodies uncover a previously unrecognized mechanism of antibody-mediated synaptic dysfunction.

## Highlights

• Synaptophysin (SYP) antibodies are found in two cerebellar ataxia patients
• Patient-derived SYP antibodies induce SYP accumulation on presynaptic membrane
• Patient-derived SYP antibodies reduce neuronal activity

## Graphical abstract

**Figure:**
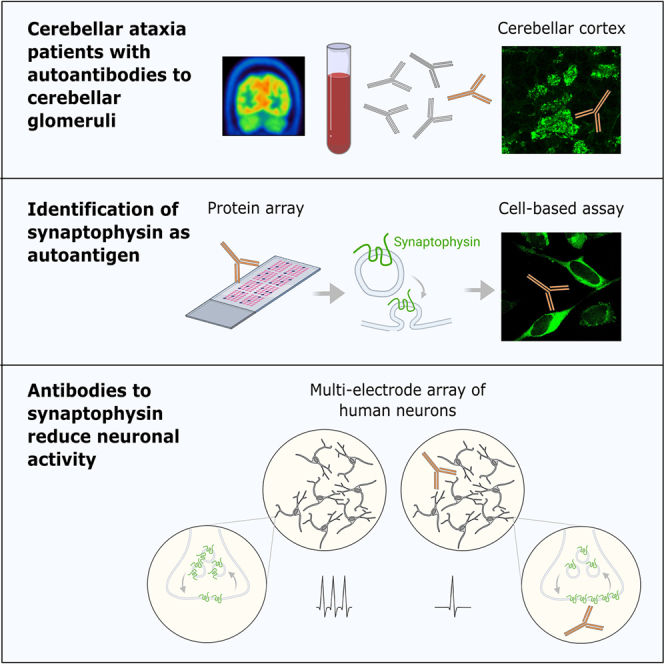

## Teaser

Ho et al. identify an autoantibody against the synaptic protein synaptophysin (SYP) in two patients with adult-onset cerebellar ataxia. They observe reduced neuronal activity and SYP mislocalization in neurons exposed to SYP-reactive antibodies. Identification of SYP antibodies and elucidation of their pathogenic activity offer potential for disease stratification and therapeutic intervention.

## Introduction

Cerebellar ataxia (CA) is a syndrome shared by many autoimmune neurological disorders and is often associated with the presence of neuron-reactive IgG,^1^^,^^2^^,^^3^ but for many patients, the precise target of these neuron-reactive antibodies has not been identified. Due to the progressive and debilitating symptoms, there is an urgent need for early and effective treatment to prevent irreversible neuronal damage. However, disease progression, available diagnostic tools, and response to treatment vary considerably among patients, likely due to different antigenic targets and their associated effector functions.^4^

Numerous antibodies identified in recent years are associated with neurological diseases. Autoantibodies targeting extracellular antigens, such as cell surface proteins or secreted ligands, are often considered pathogenic due to their potential to disrupt protein function, signaling pathways, or induce antibody-mediated cell death.^4^^,^^5^

Understanding the clinical phenotypes associated with autoantibodies and the mechanisms of antibody-mediated damage is crucial for disease classification and the development of effective treatments.^6^^,^^7^ This has been, for example, demonstrated by the identification of autoantibodies to contactin-associated protein-like 2 (Caspr2),^8^^,^^9^^,^^10^ N-methyl-D-aspartate receptor (NMDAR),^11^^,^^12^ leucine-rich glioma-inactivated 1 (LGI1) antibody,^13^^,^^14^ and metabotropic glutamate receptor 5 (mGluR5).^15^ However, many CA cases remain idiopathic.^16^ Further understanding of the disease etiology, including the identification of undiscovered autoantigens, is essential for improved diagnostic accuracy and prognostication in adult and also in pediatric^17^ patients suffering from this severe disease.

In this study, we report the discovery and characterization of synaptophysin (SYP) autoantibodies in CA. This was initially identified through strong serum autoantibody reactivity against primate cerebellar tissue in a patient with idiopathic CA (index patient 1), who tested negative for well-characterized disease-associated antibodies. Protein array screening, western blot (WB) with cerebellar membrane lysates, and a cell-based assay (CBA) identified SYP as the target antigen. SYP is a highly abundant protein expressed on synaptic vesicles that are mostly located intracellularly, but the two intraluminal loops of SYP (amino acid region 50–106 and 162–199) become exposed to the extracellular surface upon fusion with the presynaptic membrane during neurotransmitter release.^18^ Mutations in *SYP* have been linked to X-linked intellectual disability with epilepsy and frontotemporal dementia.^19^^,^^20^^,^^21^ After we validated SYP as an autoantigen of index patient 1, we developed a high-throughput CBA and screened serum samples of 143 patients with sporadic CA, of which 43 had shown synaptic tissue reactivity. These samples were derived from the prospective, longitudinal, multi-centre cohort of consecutive patients with sporadic adult-onset non-hereditary ataxia (SPORTAX cohort),^22^^,^^23^^,^^24^ as well as from individual centers in Germany and Austria. Our CBA showed that 2/43 patients with CA and a synaptic staining pattern of unknown reactivity recognized SYP.

Next, we went on to study the functional properties of autoantibodies to SYP. Since the antibodies from index patient 1 do not recognize rodent SYP, we used human induced pluripotent stem cells (hiPSCs) and found that SYP antibodies alter SYP surface retention and reduce neuronal activity, as seen with a multi-electrode array (MEA).

This study broadens the spectrum of autoantibody-associated cerebellar synaptopathies and demonstrates how anti-SYP autoantibodies impair neuronal function.

## Results

### IgG from index patient 1 recognizes excitatory presynaptic termini in cerebellar glomeruli and binds to a 38 kDa protein in cerebellar membrane lysates

**Figure 1:**
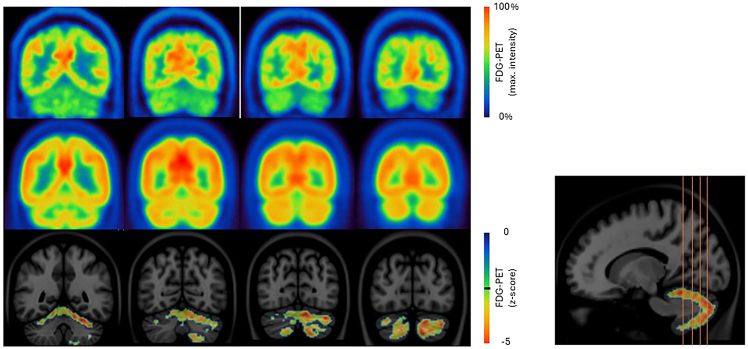
Reduced cerebellar glucose metabolism in index patient 1 on brain fluorodeoxyglucose (FDG)-positron emission tomography (PET) Coronal views of FDG-positron emission tomography (PET) imaging are shown for index patient 1 (top row), compared with a coronal view of a brain FDG-PET template (average of a healthy cohort) (middle row). An overlay of the coronal view of brain FDG-PET and template magnetic resonance imaging (MRI) displaying *Z* scores of index patient 1 versus template (displayed *Z* score: −2,5 to −5) shows a clear reduction in glucose metabolism in the bilateral cerebellar hemispheres (left > right) (lower row). The right panel shows the corresponding layers in sagittal view. See also Figure S7.

**Figure 2:**
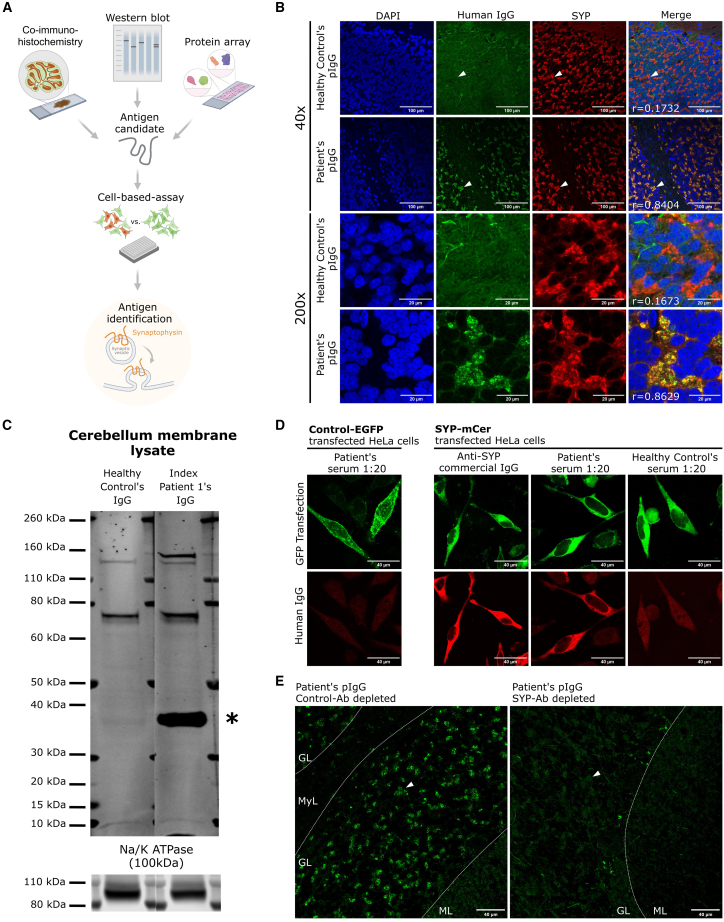
Identification of SYP as an autoantigen in CA (A) Strategy of autoantigen identification. (B) Co-immunohistochemistry of healthy control’s and index patient 1’s purified IgG (green; 400 μg/mL) with commercial anti-SYP monoclonal antibodies (red; 1:100; Bio Techne) and 4′,6-diamidino-2-phenylindole (DAPI) (blue) on primate cerebellar tissue. Colocalization between green and red images were quantified using Pearson’s coefficient (*r*), calculated with ImageJ Colocalization Finger plug-in (*r* = 1: complete colocalization; *r* = 0: absence of colocalization). Scale bars represent 100 μm in the top two rows and 20 μm in the bottom two rows. (C) Western blot of healthy control and index patient 1’s purified IgG to human cerebellar membrane lysate under non-reducing conditions. Na/K ATPase is shown as a loading control for the membrane lysate between lanes. (D) Permeabilized CBA of healthy control’s and index patient 1’s serum, as well as a commercial anti-SYP antibody, with HeLa cells transfected with human CD2-EmGFP (control protein) or SYP-mCer (antigen). Scale bars, 40 μm. (E) IgG from index patient 1 was absorbed with HeLa cells expressing SYP or a control antigen (Ag; CD2). Subsequently, these IgG fractions were used to stain primate cerebellar tissue. Figure created with BioRender.com and Inkscape. GL, granular layer; MyL, myelin layer; ML, molecular layer. Scale bars, 40 μm. See also Figures S1–S4.

Serum and cerebrospinal fluid (CSF) samples were obtained from a 48-year-old patient with progressive degenerative adult-onset CA (index patient 1; Figures 1 and S7, Table S3, and Methods S1), who tested negative for reactivity against common disease-associated autoantigens as part of routine clinical screening. On primate cerebellar tissue, the patient’s serum and purified IgG displayed prominent reactivity to cerebellar glomeruli, the synaptic structures within the granular layer (Figures 2, S1, and S2). The patient’s IgG bound specifically to both primate and human cerebellum but showed no reactivity to rodent tissue (Figure S3D). The reactivity to primate cerebellar glomeruli is dominated by the IgG1 subclass (Figure S3B) and was absent in the serum of 60 healthy controls (Figure 2B). Furthermore, the patient’s serum IgG reactivity colocalized with anti-vGLUT1/2, suggesting that the antigen resides in excitatory presynaptic terminals (Figure S1).

WB analysis of human cerebellar membrane lysate showed IgG binding to a 38 kDa antigen under non-reducing conditions (Figure 2C).

### Identification and validation of SYP as an autoantigen

To identify the antigen responsible for the synaptic IgG staining pattern, the HuProt v4.0 protein array, containing more than 21,000 human proteins, was used to generate a list of putative candidates (Table S1). SYP (UniProt ID: P08247), candidate 34, appeared to be the most plausible candidate based on its localization to presynapses, its molecular weight (MW) of 38 kDa, and its transient exposure on the cellular surface, allowing its targeting by autoantibodies with possible pathogenic effects.^20^ Further, the patient’s IgG colocalized with a commercial anti-SYP antibody on primate cerebellar tissue (Figures 2B and S4).

SYP was confirmed as the molecular target of the antibody from index patient 1 through a CBA, conducted under fixed and permeabilized conditions. The patient’s IgG exclusively bound to HeLa cells overexpressing human SYP fused with an mCerulean tag on the N terminus (mCer-hSYP) and did not bind HeLa cells overexpressing an irrelevant transmembrane protein (CD2) (Figures 2D and S5A). Furthermore, SYP expressed by HeLa cells was specifically bound by the patient’s serum IgG but not by serum IgG from a healthy control (Figures 2D and S5A). Consistent with previous immunohistochemistry (IHC) and WB results, the patient’s IgG did not recognize murine SYP in the CBA (Figure S3C). In addition to serum, we also observed weak reactivity of the patient’s IgG in CSF (index patient 1) on mCer-hSYP-expressing HeLa cells (Figure S3A). The serum antibody titers remained stable (1:2560) over a 2-year period, except for a transient decrease after plasmapheresis (1:320), indicating ongoing antibody production throughout the disease course. Serial titrations of IgG reactivity from index patient 1 and index patient 2, compared to a healthy control on CBA, are shown in Figure S5B.

Anti-SYP antibody depletion abolished IgG reactivity to primate cerebellar glomeruli, while the control-depleted IgG retained its SYP IgG reactivity (Figure 2E), confirming that anti-SYP antibodies mediated the observed synaptic IgG reactivity. The depletion of SYP antibodies was further validated by loss of IgG reactivity to SYP-expressing HeLa cells in a CBA, whereas control-depleted fractions retained their reactivity (Figure S6A).

### Screening of patient cohorts for anti-SYP antibodies

**Figure 3:**
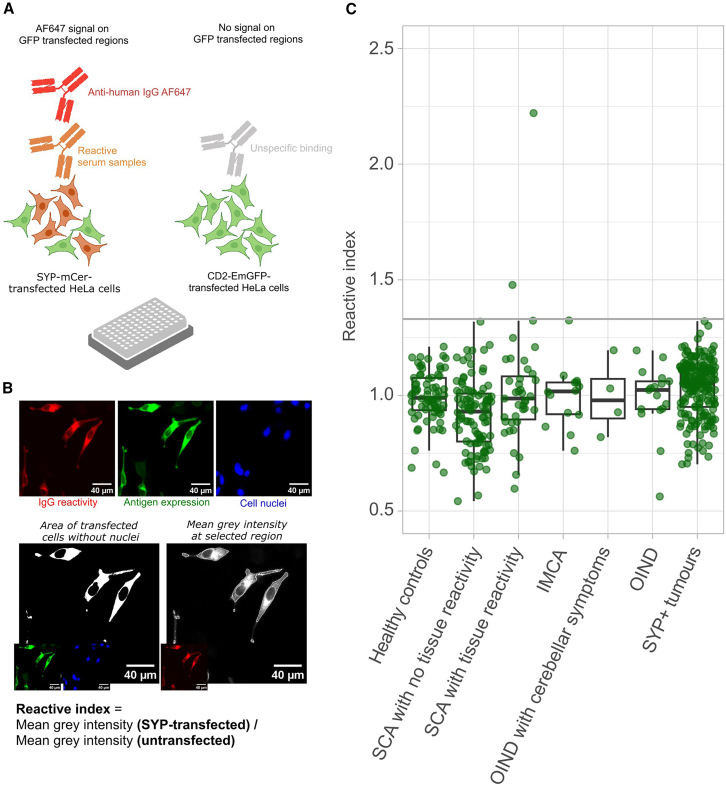
Identification of two patients with antibodies against human SYP (A) CBA scheme. (B) Image analysis workflow following CBAs to screen patients’ sera for reactivity against SYP-transfected HeLa cells. (C) Quantification of images from CBA screening of cohorts, including healthy controls (*n* = 60), sporadic cerebellar ataxia (SCA) without known etiology and without primate or rodent tissue IgG reactivity (*n* = 100), sporadic cerebellar ataxia (SCA) without known etiology with synaptic primate or rodent tissue IgG reactivity (*n* = 43), immune-mediated cerebellar ataxia with antibodies to GAD65, septin-5, amphiphysin, and Rho (IMCA; 15), other inflammatory neurological diseases (OIND) with cerebellar symptoms (*n* = 4), OIND (*n* = 28), and patients with SYP-expressing tumors (*n* = 195). Boxplot are represented as median with interquartile range. The cut-off was defined as three standard deviations above the mean reactive index of the healthy control cohort. Figure created with BioRender.com, R, and Inkscape. See also Figure S5.

To investigate the prevalence of anti-SYP antibodies among cases with synaptic reactivity, we examined a larger cohort of 143 individuals with CA of unknown etiology and 60 healthy controls for serum IgG synaptic reactivity to primate cerebellar tissue (Table S2). A subset of the CA samples (*n* = 43) was previously determined to have serum reactivity to rodent tissue. If available, we also assessed CSF IgG binding to tissue (Table S2). Synaptic IgG reactivity on primate cerebellar tissue revealed positive results in 43 individuals with CA but in none of the healthy controls. Among the 43 patients with CA with synaptic IgG reactivity on primate cerebellar tissue, two (4.7%) tested positive for serum anti-SYP antibodies using CBA (Figures 3 and S5A). No anti-SYP antibody reactivity was detected in the 100 patients with CA lacking synaptic reactivity on tissue, 15 patients with CA with identified antibodies (e.g., glutamic acid decarboxylase [GAD65], septin-5, amphiphysin, and Rho), 32 patients with other inflammatory neurological diseases, including patients with OIND selected for cerebellar symptoms (*n* = 4) or the 60 healthy controls. In addition, four serum samples and one CSF sample without clinical information were analyzed because they showed synaptic staining indicative of unknown autoreactivity (obtained from the Mayo Clinic Neuroimmunology Laboratory); however, they tested negative for SYP autoreactivity in our assay.

To assess potential intrathecal production of anti-SYP IgG, we analyzed CSF and serum samples from both index patients. In index patient 1, the albumin ratio was elevated, indicating a compromised blood-brain barrier, while the IgG ratio remained within the normal range, suggesting no clear evidence of intrathecal IgG synthesis.^25^ Anti-SYP IgG was present in the CSF, but at a titer (1:2) more than 200-fold lower than in serum (1:2560), consistent with passive diffusion rather than local production.^26^ In index patient 2, no anti-SYP IgG reactivity was detected in the CSF. Further clinical details of the two index patients are found in Figure S7, Table S3, and Text S1.

#### Patients with tumors expressing SYP in our cohort do not have antibodies to SYP in serum

Considering that SYP is ectopically expressed in tumors, such as small cell lung cancer,^27^ we also investigated whether exposure to ectopic SYP expression may contribute to the initiation of paraneoplastic anti-SYP antibody production. To understand the link between anti-SYP antibodies and ectopic SYP expression, we tested a large cohort of patients (*n* = 195) with neuroendocrine tumors or small-cell lung cancer that tested positive for ectopic SYP expression. We did not identify anti-SYP IgG in any of these patients (Figure 3).

Along this line, no tumor was detected in our anti-SYP IgG-positive index patients after thorough clinical investigation, and hence a paraneoplastic etiology is unlikely; however, this cannot be ruled out, since neurological symptoms might precede tumor detection in paraneoplastic neurological syndromes. Further investigations of the anti-SYP-producing cells in the periphery are necessary to elucidate the origin of antibody production against SYP.

### Patient’s IgG specifically recognized SYP on the surface of hiPSC-derived glutamatergic neurons

To assess the potential pathogenicity of anti-SYP antibodies, we utilized hiPSCs with rostral CNS identity differentiated into glutamatergic neurons using deterministic cell programming, following an approach first introduced in 2017.^28^

**Figure 4:**
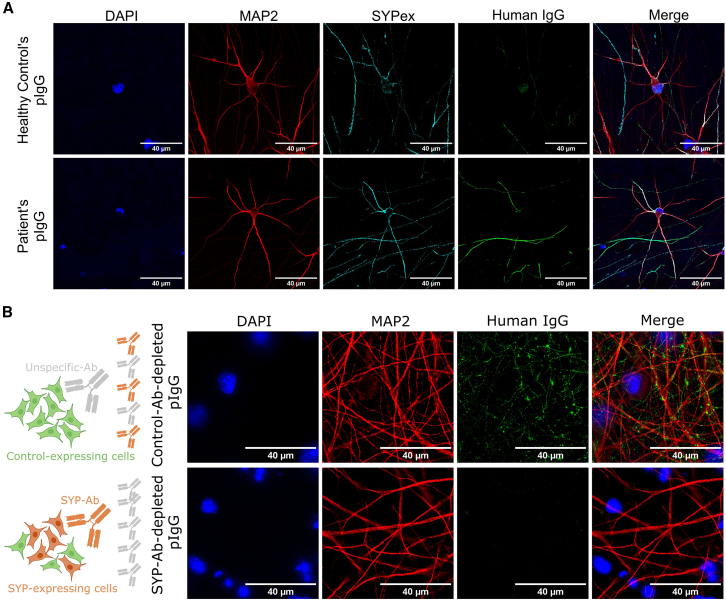
Patient’s IgG binds to SYP on hiPSC-derived glutamatergic neurons (A) Co-immunocytochemical staining of healthy control and index patient 1′s purified IgG (green; 400 μg/mL), along with MAP2 (red; 1:2000; Abcam; ab5392), commercial anti-SYP monoclonal antibody (cyan; 1:100; Biotechne; MAG5555), and DAPI (blue) on hiPSC-differentiated glutamatergic neurons under non-permeabilized conditions. Scale bars, 40 μm. (B) Cartoon of depletion of patient IgG using SYP-expressing or control-expressing cells (left). Co-immunocytochemistry of control- or reactive antibody-depleted fractions of index patient 1’s purified IgG (green; 400 μg/mL), with MAP2 (red; 1:2000; Abcam) and DAPI (blue), on hiPSC-differentiated glutamatergic neurons under non-permeabilized conditions (right). Scale bars, 40 μm. See also Figure S6.

Index patient 1’s IgG recognized endogenous SYP expression in non-permeabilized hiPSC-derived glutamatergic neuronal culture, suggesting that the IgG binds to SYP epitopes exposed on the surface (Figure 4A). The glutamatergic identity of the neurons is confirmed by the expression of vGLUT2 (Figure S6B). Under the same non-permeabilized conditions, antibodies against beta-tubulin, an intracellular antigen, showed no reactivity (Figure S8A), confirming membrane integrity and the surface specificity of our SYP staining on non-permeabilized cultures. Furthermore, SYP-depleted IgG from index patient 1 showed no reactivity compared to control-depleted IgG, demonstrating the specificity of the IgG for SYP in these cultures (Figure 4B).

### Index patient 1’s IgG and anti-SYP mAb resulted in increased SYP localization on the presynaptic membrane

**Figure 5:**
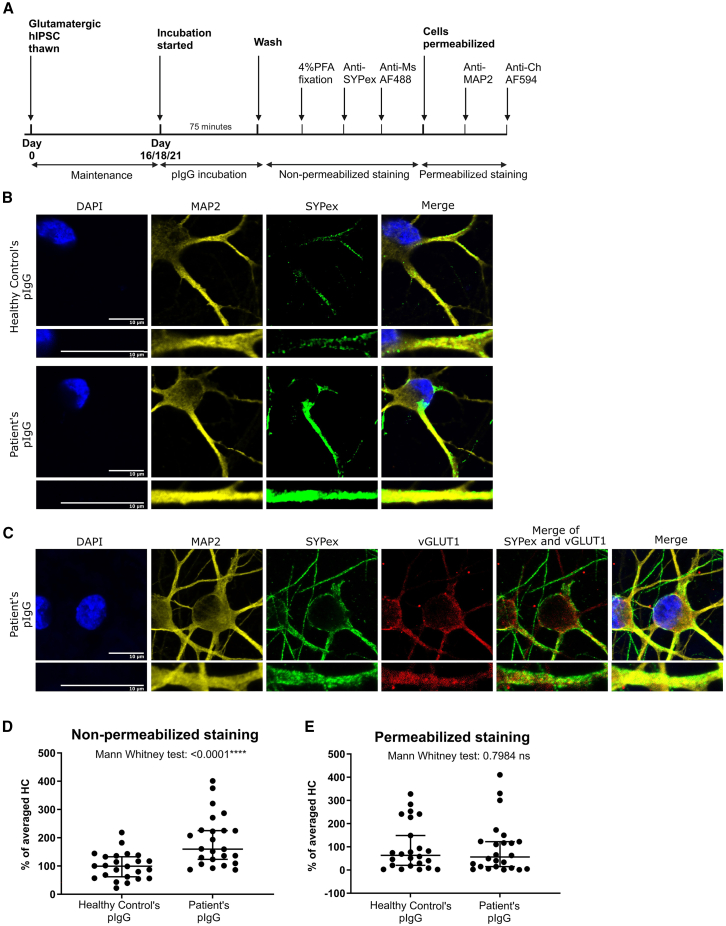
Patient’s IgG increased SYP localization on the surface in hiPSC-derived glutamatergic neurons (A) Experimental scheme. (B) Immunocytochemical detection of extracellular SYP using commercial anti-SYP antibody (green), alongside MAP2 (yellow) and DAPI (blue), following incubation of hiPSC-differentiated glutamatergic neurons with healthy control’s purified IgG or index patient 1’s purified IgG, For each condition, a higher-magnification image and a zoomed-in view of the boxed region (bottom row) are shown, with their indicated scale bars representing 10 μm. (C) Co-immunocytochemical staining of anti-SYP under non-permeabilized conditions with the presynaptic marker anti-vGLUT1 under permeabilized conditions. A higher-magnification image and a zoomed-in view of the boxed region (bottom row) are shown, with their indicated scale bars representing 10 μm. (D) Quantification of surface SYP levels on hiPSC-derived glutamatergic neurons following incubation with purified IgG from healthy control and index patient 1 (data are presented as median with 95% confidence interval (CI); each dot represents one of eight regions sampled in a well; *p* < 0.001, Mann-Whitney test). (E) Quantification of total SYP levels on hiPSC-derived glutamatergic neurons following incubation with purified IgG from healthy control and index patient 1 under permeabilized conditions (data are shown as median with 95% CI; each dot represents one of eight regions sampled in a well; *p* = 0.7984, Mann-Whitney test). ∗*p* < 0.05, ∗∗*p* < 0.01, ∗∗∗*p* < 0.001. Figure created with BioRender.com, Graphpad, and Inkscape. See also Figures S8 and S9.

Next, we investigated whether incubation of glutamatergic neurons with the patient’s IgG would alter SYP expression. Under non-permeabilized conditions, incubation with the patient’s IgG led to increased SYP surface expression compared to cells exposed to IgG from a healthy control (Figures 5B, 5D, and S9A). This extracellular SYP expression observed under non-permeabilized condition co-localizes with vGLUT1 expression detected under permeabilized conditions, confirming presynaptic localization (Figure 5C). We observed increased SYP only under non-permeabilized conditions, while there was no alteration when the staining was performed under permeabilized conditions (Figures 5E and S9B), showing that the presence of patient anti-SYP antibodies affected only the localization of SYP on the surface and not the total SYP expression.

### Index patient 1’s IgG and anti-SYP mAb resulted in reduced synaptic activity

**Figure 6:**
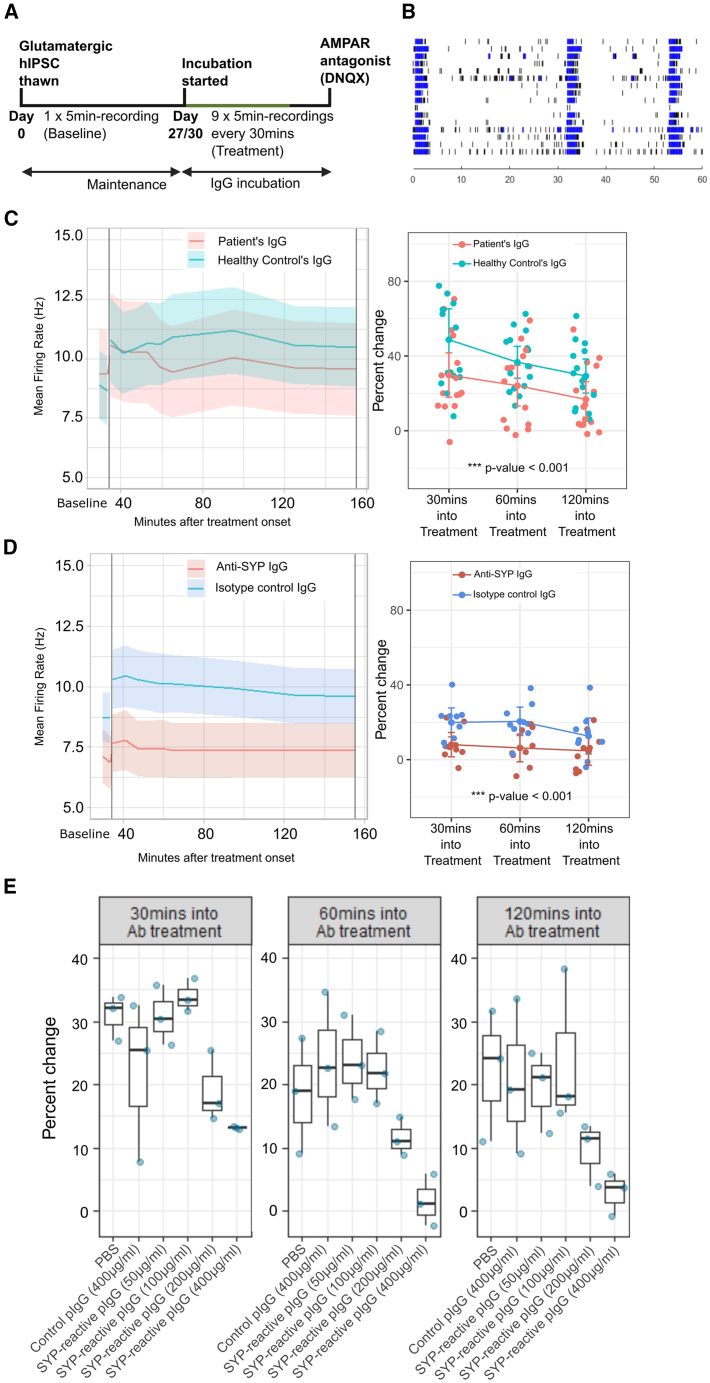
Patient’s IgG reduced populational neuronal activity in hiPSC-derived glutamatergic neurons (A) Experimental scheme of MEA recordings of hiPSC-derived glutamatergic neurons. (B) Raster plot of a representative well with 16 electrodes (rows) recording from hiPSC-derived glutatmatergic neurons at day 27 before treatment over a 60-s interval using Axion Neural Metric Tool. Black bars indicate spikes, and blue bars indicate bursts. Bursts are defined as events in which a minimum of 5 spikes occur with an inter-spike-interval below 100 ms. (C) Mean firing rate (Hz) of hiPSC-derived glutamatergic neurons incubated with index patient 1’s (red) or healthy control’s (blue) purified IgG (400 μg/mL) at baseline and over time in minutes starting 30 min after treatment application. On the right, a linear mixed model was used to determine the effect of antibody treatments on the number of spikes at 30, 60, and 120 min into the treatment (data are shown as 95% confidence interval; each dot represents one well; *p* < 0.001, linear mixed model). (D) Mean firing rate (Hz) of hiPSC-derived glutamatergic neurons incubated with SYP-reactive IgG2b monoclonal antibody (red) or isotype control IgG2b antibody (blue) (20 μg/mL) at baseline and over time in minutes starting 30 min after treatment application. On the right, a linear mixed model was used to determine the effect of antibody treatments on the number of spikes at 30, 60, and 120 min into the treatment (data are shown as 95% confidence interval; each dot represents one well; *p* < 0.001, linear mixed model). ∗*p* < 0.05, ∗∗*p* < 0.01, ∗∗∗*p* < 0.001. (E) Dose-dependent effect of SYP-reactive IgG on neuronal activity: Percent change in mean firing rate normalized to baseline of the same well in hiPSC- derived glutamatergic neurons at 30 min (left), 60 min (middle), and 120 min (right) into antibody treatment. The culture was treated with either PBS, healthy control’s purified IgG at 400 μg/mL, or index patient 1’s purified IgG (50, 100 , 200, or 400 μg/mL). Each data point represents a well. The boxplot shows the median and interquartile range for each condition. Figure created with R and Inkscape. See also Figure S10.

Next, we evaluated the impact of SYP-IgG on neuronal transmission using MEA recordings (Figure 6A). Baseline recordings conducted for 5 min directly prior to treatment application confirmed spontaneous and synchronous activity in hiPSC-derived glutamatergic neuronal cultures (Figures 6B and S10). Following a 30-min incubation, the time point at which we previously observed increased surface SYP expression, patient SYP-reactive IgG significantly reduced the mean firing rate compared to neurons incubated with healthy control IgG and vehicle control (Figures 6C and 6D). This effect was evident after 30 min of incubation, the time point at which we previously observed increased SYP surface expression and persisted for at least 120 min. Moreover, we observed a dose-dependent effect on the reduced number of spikes during antibody treatment (Figure 6E). A similar pattern of reduced mean firing rate was evident when we repeated this experiment with monoclonal mouse anti SYP IgG2b but not with isotype monoclonal IgG2b further validating our data.

Altogether, these findings demonstrate that SYP-Abs have a reducing effect on the firing rate of hiPSC-derived glutamatergic neurons. Lastly, neuronal network activity desynchronized after treatment with DNQX, indicating that the culture comprised mainly glutamatergic neurons (Figure S10).

## Discussion

In this study, we detected SYP as an autoantigen in two patients with CA and showed, in functional experiments with hiPSC-derived neurons, that autoantibodies to SYP reduce neuronal activity. The detection of SYP antibodies originated from index patient 1 with idiopathic CA, who showed strong synaptic IgG reactivity on primate cerebellar tissue while testing negative for known disease-associated antibodies. Subsequent protein array analysis and confirmation by a CBA and absorption experiments confirmed SYP as the target autoantigen.

Serum antibodies from index patient 1 detected SYP in transfected HeLa cells only under permeabilized conditions, although we observed reactivity to surface SYP on hiPSC-differentiated glutamatergic neurons. While cycling of SYP to the surface is established in neurons, it is unclear whether HeLa cells are also able to do this. Therefore, we used permeabilized HeLa cells to establish an automated screening system.^29^

This high-throughput CBA allowed us to screen serum samples from 143 patients with idiopathic CA that were derived from the prospective, longitudinal, multi-centre cohort of consecutive patients with sporadic adult-onset non-hereditary ataxia (SPORTAX cohort),^22^^,^^23^^,^^24^ as well as from individual centers in Germany and Austria. We recruited a subset of samples (*n* = 43) in which serum reactivity to rodent tissue of unknown reactivity was previously reported, yet not exclusive to synapses, and with no specific autoantibody identified. Therefore, the percentage of CA samples with tissue synaptic reactivity may not represent the extent of synaptic reactivity in CA samples reported by other groups. For this, a previous study observed neurophil reactivity to rodent tissue in 17% of patients with sporadic CA.^30^ Importantly, in our study, SYP antibodies were detected in 2/43 patients with sporadic CA without known etiology who had synaptic tissue reactivity.

We did not detect SYP antibodies in patients with CA of unknown etiology who lacked synaptic serum reactivity (*n* = 100) nor in patients with CA with identified target antibodies^31^^,^^32^^,^^33^^,^^34^^,^^35^^,^^36^ (e.g., GAD65, septin-5, amphiphysin, and Rho; *n* = 15) nor in patients with OIND, including those selected for CA symptoms (*n* = 4). Moreover, we also did not detect anti-SYP antibodies in patients with tumors that ectopically express SYP (*n* = 195), including small-cell lung cancer (*n* = 193) and neuroendocrine tumors (*n* = 2). Previously, weak serum reactivity to SYP was detected in one patient with small-cell lung cancer with paraneoplastic neurological symptoms.^37^ As neurological examinations were not routinely performed in our small-cell lung cancer cohort, we could not comment on the proportion of patients with paraneoplastic neurological symptoms in our cohort. Future recruitment and screening for serum anti-SYP antibodies in patients with small-cell lung cancer and paraneoplastic neurological symptoms will be crucial for understanding the prevalence and roles of anti-SYP antibodies in paraneoplastic contexts.

We identified two male patients with CA who tested positive for SYP-specific IgG. One patient also exhibited mild cognitive impairment, potentially indicating a broader synaptopathy, as SYP is expressed throughout the brain. Similar cognitive features have been reported in ataxia with mGluR1 antibodies.^38^^,^^39^ To our knowledge, these are the only reported cases of anti-SYP IgG-associated ataxia, highlighting a potentially undiscovered autoimmune phenotype. We recommend including anti-SYP antibody testing in the diagnostic workup of suspected autoimmune CA.^40^

Although immunotherapies, including plasma exchange and B cell-depleting therapy, did not improve disease in index patient 1, this lack of response may reflect irreversible neuronal damage rather than treatment inefficacy,^41^ highlighting the importance of early diagnosis and intervention.^41^ Future studies are needed to further define the clinical spectrum and treatment responsiveness of SYP autoimmunity. For instance, insights into the effector mechanisms of anti-SYP autoantibodies may inform the potential efficacy of therapies targeting complement-dependent cytotoxicity and FcyR-dependent cytotoxicity. Further identification of patients harboring anti-SYP autoantibodies will be critical to understanding the clinical phenotypes and the underlying autoantibody-mediated pathophysiology.

Although anti-SYP reactivity was observed in the CSF of patient 1, our comparative CSF-serum did not show evidence of intrathecal IgG production against SYP, indicating a peripheral origin of anti-SYP antibodies, similar to cases of other CNS-reactive autoantibodies, such as those typically seen in anti-MOG and anti-AQP4 antibodies in MOG antibody-associated disease (MOGAD) and neuromyelitis optica spectrum disorder (NMOSD), two inflammatory diseases of the CNS.^42^ Those autoantibodies produced in the periphery may enter the CNS upon breakdown of the blood-brain-barrier (BBB) integrity due to other conditions, such as systemic inflammation.^43^ In addition, low levels of antibodies may enter the CNS passively. This passive diffusion of peripheral antibodies into the CNS has been demonstrated by the success of intravenous monoclonal antibody (mAb) treatments against CNS antigens, such as lecanemab.^44^ Although our data do not support intrathecal synthesis of anti-SYP IgG in the two index cases, it is a limitation that we did not have access to CSF samples from the broader patient cohort. Therefore, further studies are needed to assess the frequency and relevance of SYP autoantibodies, including CSF and serum.

Our identification of SYP as an autoantigen increases the possibility to stratify further patients with CA. Patients with autoantibodies to CNS antigens show a broad range of clinical symptoms and disease courses,^7^ and patients with SYP antibodies will be added to this spectrum. The relevance of anti-SYP autoantibodies may extend beyond patients with CA, as they were recently detected in individuals with occupational autoimmune inflammatory polyradiculoneuropathy with neuropathic pain and demyelination.^45^ We found that autoantibodies to SYP bind to the surface of human neurons. Therefore, patients with anti-SYP antibodies can be added to the heterogeneous group of patients with antibodies to neuronal surface structures.

**Figure 7:**
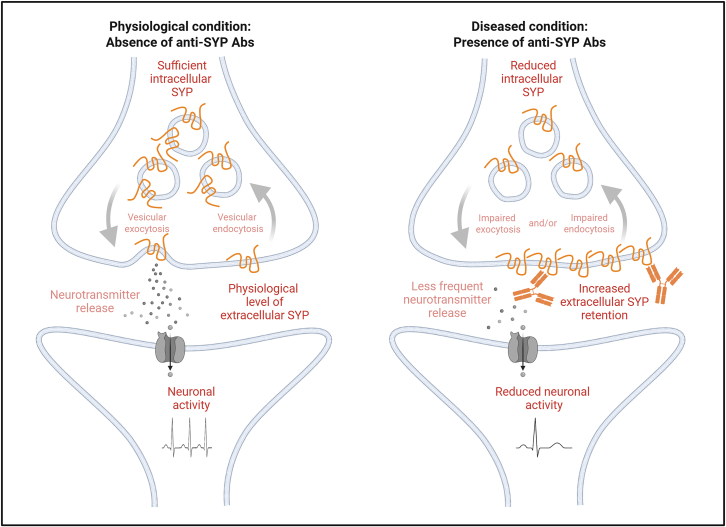
Proposed pathogenic mechanism of anti-SYP antibodies at neuronal synapses Fonts in dark red indicate obtained findings. Text written in light red represents proposed events. In the presence of anti-SYP antibodies, a higher level of SYP is retained on the presynaptic membrane and less intracellular SYP is observed. This may be the consequence of impaired vesicular endocytosis and may result in dysfunctional exocytosis that can potentially lead to less frequent neurotransmitter release, explaining the observed reduced neuronal activity recording in our MEA data. Figure created with Biorender.com.

Our functional experiments demonstrated that anti-SYP autoantibodies mediate surface retention of SYP in hiPSC-derived neurons and reduce neuronal activity. This represents a previously undescribed mechanism of autoantibody-mediated CNS dysfunction. Electrophysiological studies in SYP knockout mice revealed that SYP has a critical role in the kinetically efficient endocytosis of synaptic vesicles, which is essential for replenishing the recycling vesicular pool following neurotransmission.^29^^,^^46^ Furthermore, observations using *in vitro* vesicular fusion assays also showed the indispensable role of SYP in accelerating fusion of synaptic vesicles with a planar membrane that simulates the presynaptic membrane, highlighting the role of SYP in vesicular exocytosis and neurotransmitter release.^29^ Indeed, SYP deletion results in reduced synaptic vesicle endocytosis, a phenotype rescued by wild-type SYP expression.^47^ The antibody-mediated retention of surface SYP observed in our study may impact synaptic vesicles retrieval, hindering the replenishment of the recycling vesicular reserve and potentially disrupting vesicle docking and fusion of synaptic vesicles during neurotransmitter release (Figure 7). We propose this model based on the above-outlined features of SYP and our MEA recordings, showing reduced neuronal activity in hiPSCs incubated with the patient’s IgG and a mouse monoclonal SYP antibody. While this suggests a link between reduced vesicle recycling and the observed increase in surface SYP, further research is needed to establish a direct causal relationship and fully elucidate the underlying mechanisms. Notably, SYP plays a role in soluble N-ethylmaleimide-sensitive fusion protein attachment protein receptor (SNARE) complex formation, which is essential for vesicular fusion with the presynaptic membrane for neurotransmitter release.^48^ Specifically, SYP interacts with synaptobrevin (SYB), competing with SYB-syntaxin complex formation to temporally regulate neurotransmitter release.^49^ We primarily attribute the observed autoantibody effects to synaptic alterations. It is possible that the increased presynaptic membrane retention of SYP may contribute to intracellular SYP depletion, ultimately diminishing neuronal activity. Indeed, prior work in *Xenopus* neurons has shown that injecting SYP-reactive antibodies into the soma decreases spontaneous synaptic frequencies and reduces the amplitude of impulse-evoked synaptic currents, suggesting impaired neurotransmitter quantal release.^50^ Potential mechanistic connections between our two observations suggest that increased surface retention of SYP could have several downstream impacts on synaptic vesicle function. It may not only impair vesicle endocytosis, leading to a depletion of the presynaptic vesicle pool available for neurotransmitter transport, but could also affect the abundance of SYP on individual vesicles, thereby impacting their docking and fusion with the presynaptic membrane. Our study did not assess changes in the presynaptic vesicle reserve or the potential interference of anti-SYP antibodies with SYP-SYB complex formation. In addition, further investigation into the kinetics of SYP retention on the presynaptic membrane will be crucial in follow-up studies for understanding the mechanisms of anti-SYP-mediated alterations in synaptic function.

Given SYP’s diverse roles in synaptic transmission, the observed SYP retention on the surface is a plausible explanation for the reduced neuronal activity in the presence of anti-SYP antibodies. However, we cannot exclude the contribution of other disrupted SYP functions to this decreased neuronal output.

While the precise antigenic epitopes of SYP targeted by the patient’s antibody remain unknown, the observed IgG reactivity to glutamatergic neurons under non-permeabilized conditions, coupled with the antibody-mediated functional effects, indicates that at least one of the two intravesicular loops exposed extracellularly upon fusion with the presynaptic membrane is involved. While the intracellular C-terminal fragment of SYP mediates the interaction with SYB, which is essential for synaptic vesicle fusion,^51^ antibody binding to the extracellular loops may induce or impede conformational changes required for this interaction.

While our current study demonstrates that the directly antibody-mediated extracellular SYP retention can lead to a functional reduction in neuronal activity, this may only partially contribute to the pathophysiology, and further investigations using *in vivo* or *ex vivo* models could recover additional inflammatory consequences of anti-SYP antibodies. Due to the lack of recognition of patient antibodies to rodent SYP, it is a limitation that no *in vivo* rodent model was feasible for functional investigations. This can be addressed once patients antibodies are identified that cross-react with rodent SYP or through more complex systems, such as *ex vivo* human tissue slices. These approaches would allow the study of longer time points of SYP antibody exposure to uncover downstream amplification or compensatory processes of antibody-mediated effects. In addition, potential effects of microglial-mediated synaptic pruning through complement activation or Fc receptor-dependent pathways can be investigated.

SYP is ubiquitously expressed in neuronal synaptic terminals, not just cerebellar neurons. Therefore, circuit-level intricacies may be crucial in understanding the differential responses of different CNS regions to anti-SYP antibodies and why clinical manifestations may predominantly involve cerebellar dysfunctions. There is a view that, whereas neuronal networks in other brain regions contain redundant parallel processes, potentially acting to compensate for local dysfunction, the cerebellar network converges highly onto the sole Purkinje cell output, leading to a more vulnerable network during synaptic dysfunction. In addition, microglia have been reported to exhibit heightened vigilance and increased damaged neuronal clearance activity, particularly in the cerebellum compared to other brain regions.^52^^,^^53^ Furthermore, the cerebellar BBB has been demonstrated to be more permeable to inflammatory stimuli.^54^ Altogether, the lack of compensatory parallel neuronal processes, heightened microglial activity, and a more permeable BBB in the cerebellum may render it more vulnerable to inflammation. Ultimately, identification of further patients with anti-SYP antibodies could help characterize unique clinical phenotypes associated with these antibodies.

The retention of a protein involved in synaptic vesicle formation and recycling represents a previously unrecognized mechanism of antibody-mediated neuronal dysfunction.^4^^,^^7^ This differs from established autoantibody effects in the CNS, which include complement activation and Fc receptor-mediated cytotoxicity (e.g., in MOG-, AQP4-, or anti-neurofascin-IgG3-associated diseases), blocking of receptor signaling (e.g., gamma-aminobutyric acid type B receptor [GABA-B] receptor autoantibodies), internalization of surface receptors (as seen in NMDAR encephalitis), interference with axo-glial interactions (anti-neurofascin NF-IgG4), interruption of binding to synaptic receptors (LGI1), or disruption of interactions of secreted ectodomains with other proteins (immunoglobulin-like cell adhesion molecule 5 [IgLON5]).^7^^,^^55^^,^^56^

We have identified SYP as a synaptic antigenic target in two patients with sporadic CA. This increases the possibility of stratifying patients with CA. The anti-SYP antibodies bound to the surface of human neurons, enhanced retention of SYP on the surface, and reduced synaptic activity, highlighting a previously undescribed mechanism of Ab-mediated CNS injury.

### Limitations of the study

We found that anti-SYP enhances retention of SYP on the membrane and reduces synaptic activity, but we cannot yet determine how exactly SYP retention and synaptic dysfunction are linked. Our study shows that anti-SYP reduces neuronal activity in cultured neurons, but we have not shown a pathogenic effect using *in vivo* models. Future studies, ideally with patient-derived mAbs that recognize rodent SYP, will provide further insight and will allow the contribution of microglia to pathogenicity to be dissected. We have detected only two patients with CA and autoantibodies to SYP so far; however, our report of these two patients and the description of an assay that can be used to identify anti-SYP autoantibodies will allow anti-SYP to be linked more precisely with clinical features.

## Resource availability

### Lead contact

Further information and requests for resources and reagents should be directed to and will be fulfilled by the lead contact, Simone Mader (simone.mader@uk-erlangen.de).

### Materials availability

Patient-derived materials originate from participating clinical centers and are controlled by the respective institutions. Access to such materials is subject to ethical approval, institutional regulations, and material availability.

Further details are available from the lead contact upon request.

### Data and code availability

• The datasets generated and analyzed during this study are available from the corresponding author on reasonable request.
• The data underlying quantification of intensities at MAP2+ pixels, representing presynapses proximal to dendrites in Figure 5, has been provided as a macroscript (supplemental information).
• Any additional information required to reanalyze the data reported in this work is available from the lead contact upon request.

## Acknowledgments

This study has been enabled by the following: the 10.13039/501100003042Else Kröner-Fresenius-Stiftung (to S.M.), the 10.13039/501100001660Schering Stiftung and the 10.13039/501100003390Fritz Thyssen Foundation (to S.M.), the 10.13039/100002243National Ataxia Foundation (to S.M.), the DFG (SFB TR128 and ME 1509/2-1 to E.M.), the Verein zur Therapieforschung für MS Kranke (to E.M.), the 10.13039/501100002428Austrian Science Fund FWF SYNABS I6565-B (R.H.), Forschungsförderungsgesellschaft FFG IGNITEMIND FO999920011 (R.H.), and the Austrian Society of Neurology (Österreichische Gesellschaft für Neurologie) (R.H., T.S-H.). The authors would like to thank Betty Diamond, Sabine Liebscher, and Florence Bareyre for comments on the manuscript. The authors gratefully acknowledge access to clinically uncharacterized serum samples with granular layer staining pattern of unknown reactivity by the Mayo Clinic Neuroimmunology Laboratory. The authors gratefully acknowledge access to the longitudinal, multi-center cohort of consecutive patients with sporadic adult-onset ataxia (SPORTAX), as well as the samples and data provided by the Lung Biobank Heidelberg, part of the Biomaterial Bank Heidelberg (BMBH), and all participating centers that contributed samples to this study, as well as all patients involved. The authors would like to thank Mike Cousin and Hisashi Umemori for providing the mCer-hSYP and rSYP-YFP plasmids, respectively. The authors are grateful to the Technology Platform Pluripotent Stem Cells of the Max Delbrück Center for providing the BIHi005-A-24 line and for their support with the MEA experiments. The authors are very grateful to bit.bio for their generous support and expertise in the human iPSC-derived glutamatergic neurons. The authors would also like to extend their gratitude to PEPperPRINT for their continued support and to Ivanela Kondova for providing rhesus tissue blocks essential for establishment of assays, as well as Andree Schmidt for assistance during antigen identification. Furthermore, the authors would like to thank Andreas Thomae and Jan Brocher for valuable advice on image analyses and Tobias Straub and Yemil Atisha-Fregoso for statistical expertise. The authors are grateful to Stefan Lichtenthaler and Eric Chang for valuable discussions. Finally, the authors appreciate the technical support by Christian Lad, Yaren Canten, Ceren Özdemir, Elif Dönmez, and the clinical staffs at the outpatient clinic of the Institute of Clinical Neuroimmuniology of LMU Munich.

## Author contributions

E.M. and S.M. conceived and directed the project. S.H., L.B, T.S.-H., R.H, S.F., L.A.G., E.M., and S.M. wrote the manuscript. All authors contributed to editing of the manuscript. S.H. performed all experiments. S.H., H.K.W., D.S., S.W., M.G.-E., K.R., T.K., F.S.T., A.V., L.B., M.A.S., T.M., T.S.-H., V.E., R.H., J.F., T.K., S.F., and L.A.G. participated in data acquisition. S.F. and S.H. conducted and analyzed the MEA data. S.H., S.F., L.B., S.M., and E.M. analyzed the data. S.F. and R.H, and L.A.G. contributed to the interpretation of findings. All authors contributed to the critical reading and editing of the manuscript. E.M. and S.M. contributed equally and share last authorship.

## Declaration of interests

The authors have declared that no conflict of interest exists. A complete list of disclosures is in the supplemental information (Methods S2).

## Declaration of generative AI and AI-assisted technologies in the writing process

During the preparation of the work, the authors occasionally used ChatGPT in order to improve the wording. After using this tool, the authors reviewed and edited the content as needed and take full responsibility for the content of the published article.

## STAR★Methods

### Key resources table

**Table:** 

REAGENT or RESOURCE	SOURCE	IDENTIFIER
**Antibodies**		

Chicken anti-MAP2	Abcam	CAT#: ab5392; RRID: AB_2138153
Rabbit anti-Na/K ATPase	Invitrogen	CAT#: MA5-32184; RRID: AB_2809472
Guinea pig anti-Synapsin 1	Synaptic Systems	CAT#: 106 004; RRID: AB_1106784
Mouse anti-SYP	BioTechne	CAT#: MAB5555; RRID: AB_3658464
Mouse anti-SYP antibody	Dako	CAT#: GA66061-2; RRID: AB_3698021
Rabbit anti-vGLUT1/2	Synaptic Systems	CAT#: 135 403; RRID: AB_887883
Rabbit anti-vGLUT1	Synaptic Systems	CAT#: 135 303; RRID: AB_887875
Donkey anti-Mouse IgG – AF488	Thermos Fisher	CAT#: A21202; RRID: AB_141607
Goat anti-Chicken IgY- AF594	Thermo Fisher	CAT#: A11042; RRID: AB_2534099
Goat anti-Human IgG - AF488	Southern Biotech	CAT#: 2049–30; RRID: AB_2795698
Goat anti-Human IgG – AF647	Thermo Fisher	CAT#: A21445; RRID: AB_2535862
Mouse anti-Human IgG1 – biotinylated	Southern biotech	CAT#: 9052–08; RRID: AB_2796620
Mouse anti-Human IgG2 – biotinylated	Southern biotech	CAT#: 9060–08; RRID: AB_2796634
Mouse anti-Human IgG3 – biotinylated	Southern biotech	CAT#: 9210–08; RRID: AB_2796700
Mouse anti-Human IgG4 – biotinylated	Southern biotech	CAT#: 9200–08; RRID: AB_2796692
Rat anti-Mouse IgG - AF647	Jackson	CAT#: 415-605-166; RRID: AB_2340285
Streptavidin IRDye 800	Licor	CAT#: 926–32230

**Bacterial and virus strains**		

NEB® 5-alpha Competent *E. coli* (High Efficiency)	New England Biolabs	C2987

**Biological samples**		

Human cerebellum whole lysate	Novus Biologicals	NB820-59180
Human cerebellum membrane lysate	Novus Biologicals	NB820-60572
EUROIMMUN Neurology Mosaic 17 (Primate cerebellar tissue sections)	EUROIMMUN	FB1111-1010-17
EUROIMMUN IIFT Glutamate Receptor Mosaik 3 (Rodent cerebellar tissue sections)	EUROIMMUN	FA111m-1003-3

**Chemicals, peptides, and recombinant proteins**		

2-Mercaptoethanol	ThermoFisher	31350010
2-Propanol (for Analysis) [(CH3)2CHOH] MW: 60.10 g/mol, CAS No. [67-63-0]	Merck	1.09634.2511
4′,6-diamidino-2-phenylindole (DAPI)	Thermo Fisher	62248
4% paraformaldehyde (PFA)	Santa Cruz Biotechnology	sc-281692
B27	ThermoFisher	17504044
BDNF	R&D	248-BDB-005
Borate buffer (20×)	ThermoFisher	28341
Bovine serum albumin (BSA)	Sigma	A3059
DAPT	Biotechne	2634
DMEM - high glucose	Sigma	D5796
Doxycycline	Sigma	D9891
Ethanol absolut, EMSURE® [C_2_H_5_OH] MW: 46,07 g/mol, CAS No.[64-17-5]	Merck	1.00983.2511
FBS Superior; standardized Fetal Bovine Serum	Merck	S0615
Fluorescence Mounting Medium	Dako	S3023
Geltrex (Reduced GF)	ThermoFisher	A1413202
Glutamax	ThermoFisher	35050061
HaltTM Protease Inhibitor Cocktail (100×)	Thermo Scientific	1862209
Methanol ≥99.9%, EMSURE® [CH_3_OH] MW: 32,04 g/mol, CAS No.[67-56-1]	Merck	1.06009.2511
Neurobasal	ThermoFisher	21103049
Normal Goat Serum	Thermo ScientificTM	16210064
Novex TM Sharp Pre-stained Protein Standard	Invitrogen	LC5800
NT3	R&D	267-N3-005
NuPAGE™ LDS Sample Buffer (4×)	Thermo fisher	NP0007
NuPAGE™ Sample Reducing Agent (10×)	Thermo fisher	NP0009
NuPAGE™ MOPS SDS Running Buffer (20×)	Thermo fisher	NP0001
NuPAGE™ Transfer Buffer (20×)	Thermo fisher	NP00061
OptiMEM (1×)	Gibco	31985–062
PBS – Phosphate-Buffered Saline	Thermo ScientificTM	10010015
PDL-hydrobromide	Sigma	P6407
Penicillin-Streptomycin (10,000 U/mL)	Gibco	15140122
Saponin	Sigma	84510
Skimmed Milk Powder	Spinnrad	2231018
Triton® X 100	Sigma Aldrich	T8787
Trypan Blue Stain (0.4%)	InvitrogenTM	T10282
Tween 20, 100% Nonionic Detergent	Bio-Rad Laboratories	1706531
UltraPure™ DNase/RNase-Free Distilled Water	InvitrogenTM	10977–035
Vectashield HardSet Antifade mounting media	VectorLab	H-1400-10

**Critical commercial assays**		

Amicon® Ultra Centrifugation filter 30 kDa MWCO	Merck	UFC9030
Human IgG ELISA	MabTech	3850-1AD-6
HuProt v4.0 Protein array	PEPperPRINT	N/A
Lipofectamine 2000	Thermo Fisher	11668019
NAb™ Protein A/G Spin Kit	Thermo Fisher	89980
Pierce™ BCA Protein Assay Kits	Thermo Fisher	23225

**Deposited data**		

Neuronal differentiation and MEA plating using an inducible NGN2 hiPSC-line	Technology Platform Pluripotent Stem Cells of the Max Delbrück Center	https://doi.org/10.17504/protocols.io.kqdg3q54ev25/v1

**Experimental models: Cell lines**		

HeLa cells	N/A	N/A
ioGlutamatergic neurons	bit.bio	io1001
hiPSC-derived glutamatergic neurons	Technology Platform Pluripotent Stem Cells of the Max Delbrück Center	hiPSC line BIHi005-A-24

**Recombinant DNA**		

mCer-hSYP	Prof. Mike Cousin (University of Edinburgh)	N/A
CD2-EmGFP	Lenti ORF clone of Human CD2 molecule, Origene	N/A
rSYP-YFP	Dr. Hisashi Umemori (Harvard Medical School)	N/A

**Software and algorithms**		

Biovoxxel 3D Box (v1.23.0)	Jan Brocher	N/A
ImageJ (v1.54g)	FIJI	https://github.com/fiji/fiji/
Inkscape (v1.2.1)	Inkscape	www.inkscape.org
Macro script for quantification of immunocytochemistry	This study	N/A
Rstudio (2024.09.1)	Posit	https://posit.co/download/rstudio-desktop/

**Other**		

PVDF membranes	GE healthcare	10600023
NuPAGETM 4–12% Bis-Tris 1mm gels	Thermo Fisher	NP0323BOX

### Experimental model and study participant details

#### Human cohort

This study included serum samples of patients with idiopathic adult-onset cerebellar ataxia (*n* = 143) that derived from the prospective longitudinal multi-centre cohort of consecutive patients with sporadic adult-onset non-hereditary ataxia (SPORTAX cohort),^22^^,^^23^^,^^24^ as well as from individual centers from Germany and Austria and from pediatric cerebellar ataxia (*n* = 3). Although great efforts were taken to exclude patients with acquired ataxia also through testing of specific anti-neuronal antibodies when possible, immune-mediate ataxia can not be ruled out in some cases. Hereditary causes were excluded in all cohorts. A subset of the samples (*n* = 43) from external collaborators were previously tested to have serum reactivity of unknown reactivity (not exclusively synaptic) to rodent CNS tissue. Furthermore, we also screened for anti-SYP antibodies in 32 patients with other inflammatory neurological diseases (OIND). OIND included patients with multiple sclerosis (MS; *n* = 14), of which 4 have prominent cerebellar symptoms, neuromyelitis optica spectrum disorders (NMOSD; *n* = 7), myelinating oligodendrocyte glycoprotein associated disorders (MOGAD; *n* = 1), myelitis (*n* = 5) and atypical CNS demyelinating diseases (*n* = 5). In addition, we analyzed serum samples from patients with tumors that ectopically express SYP (*n* = 195), including small-cell-lung-cancer (*n* = 193) and neuroendocrine tumors (*n* = 2), as well as from healthy controls (*n* = 60). CSF samples were available from 9 patients with cerebellar ataxia and 1 idiopathic intracranial hypertension patient.

All participants or their legal guardians provided written informed consent, and the study was conducted in accordance with the Declaration of Helsinki. Ethical approval and biological samples were obtained from the following institutions: 101 samples from Ludwig-Maximilians-Universität München, Germany (ethics approvals 24–0286 and 20–0996), 100 samples from the German Center for Neurodegenerative Diseases (ethics approval SPORTAX 621/2010BO1), 37 samples from the Medical University of Vienna, Austria (ethics approvals EK 1123/2015 and 1636/2019), 6 samples from Koç University, Türkiye (ethics approval: 2018.048.IRB2.011) and 3 pediatric samples from the Children’s Hospital Datteln, Germany (ethics approval 164/2014). Furthermore, 193 samples were provided by the Lung Biobank Heidelberg, a member of the Biomaterial Bank Heidelberg (BMBH), in accordance with biobank regulations and with approval from the ethics committee of Heidelberg University (ethics approval S-270/2001). Extensive clinical and laboratory autoantibody testing was performed on two index patients, and plasma samples from selected individuals were stored for further analysis. Samples from males and females were included in the study cohort (Table S2).

#### Mammalian cell culture

HeLa cells (human, female) were cultured in Dulbecco’s Modified Eagle Medium (DMEM) (D5796; Sigma) supplemented with 10% (v/v) heat-inactivated fetal bovine serum (FBS) (S0615; Sigma), 1% (v/v) Penicillin-Streptomycin (15140122, Gibco) at 37°C with 5% CO2. Cells were passaged when they reached 80% confluency using Trypsin-EDTA solution (59417C; Sigma).

#### Human IPSC derived glutamatergic neurons

Two sources of human IPSC derived glutamatergic neurons were included in this study: ioGlutamatergic neurons (Cat. no. io1001, bit.bio, Cambridge, United Kingdom) and glutamatergic neurons differentiated in-house from cell line BIHi005-A-24 at the Stem Cell Technology Platform of the Max Delbrück Center (MDC). Both differentiation utilized deterministic cell programming^28^ and have rostral CNS identity. ioGlutamatergic neurons were cultured according to manufacturer’s instructions at 37°C with 5% CO2 and maintained for 28 days in immunocytochemistry experiments. Cells were seeded at 30,000 cells/cm^2^ upon revival from cryopreservation in 24-well-plate format on glass cover slips coated with PDL and Geltrex as instructed in the user manual.

Neurons generated from the hiPSC line BIHi005-A-24 were seeded at a density of 50,000 cells on CytoView MEA 48 with 16 electrodes per well according to the published protocol: https://doi.org/10.17504/protocols.io.kqdg3q54ev25/v1 and maintained until they exhibited stable spontaneous synchronous electrophysiological activity (within 3–4 weeks after MEA seeding) at 37°C and 5% CO2.

### Method details

#### IgG purification

Polyclonal IgG were purified from patients’ plasma isolated from whole blood or from the first plasmapheresis treatment using NAb Protein A/G Spin Kit (Thermo Fisher), following the manufacturer’s instruction. Subsequently, the elution buffer was exchanged to PBS using Amicon Ultra Centrifugation filter 30 kDa MWCO (Merck). IgG concentration was determined using a microvolume spectrophotometer (Nanodrop), Pierce BCA Protein Assay Kits (Thermo Fisher) and a human IgG ELISA (MabTech). The IgG integrity was confirmed with Coomassie staining following gel electrophoresis.

#### Immunohistochemistry

To detect IgG reactivity of patients’ samples, immunohistochemistry was performed on primate cerebellar tissue sections (EUROIMMUN Medizinische Labor Diagnostika AG) using a serum dilution of 1:20 or purified IgG concentration (ranging from 50 to 400 μg/mL) diluted in PBS with 0.05% Tween 20. In order to investigate co-localization of patient IgG with commercially available SYP antibodies, samples were co-incubated with anti-SYP antibody (Dako; 1:100) at room temperature for 30 min before detecting the primary antibodies with anti-human AF488 (Southern Biotech; 1:400) and anti-mouse AF647 (Jackson Immunoresearch; 1:400). The slides were washed with PBS containing 0.05% Tween 20 for 5 min following each antibody incubation. Lastly, the slides were stained with 4′,6-diamidino-2-phenylindole (DAPI; Thermo Fisher; 1:25000) and mounted with glycerol under a coverslip.

For determining the IgG subclass of the reactive antibodies, the following biotinylated antibodies were employed: mouse-*anti*-human IgG1 (Southern biotech; 1:250), mouse-*anti*-human IgG2 (Southern biotech: 1:250), mouse-*anti*-human IgG3 (Souther Biotech; 1:250), mouse-*anti*-human IgG4 (Southern biotech; 1:250) and Streptavidin 488 (Thermo Fisher; 1:400).

#### Western blot

Human cerebellum membrane lysates (10 μg; Novus Biologicals) without heating nor reducing agent were subjected to gel electrophoresis alongside a Novex TM Sharp Pre-stained Protein Standard (Invitrogen; LC5800) using NuPAGE 4–12% Bis-Tris 1mm gels (Thermo Fisher) and mini gel tank (Thermo Fisher) before transferring to PVDF membranes (GE healthcare) using a mini blot module (Thermo Fisher) for 2 h at 30mV. The membranes were blocked with 5% milk in PBS with 0.05% Tween 20 overnight at 4°C on a shaker. Subsequently, the membranes were stained with index patient 1’s IgG and detected with anti-human IgG1 (Southern Biotech) and Streptavidin IRDye 800 (Licor; 1:2500). All antibodies were used in blocking buffer. The membranes were imaged using the Odyssey DLx Imaging System (Licor).

#### HuProt v4.0 proteome array

The HuProt Array was performed using index patient 1’s IgG purified from plasma and evaluated by the custom support at PEPperPRINT as previously reported.^57^^,^^58^ After pre-swelling in washing buffer for 15 min and a 30-min incubation in blocking buffer, the HuProt Human Proteome Microarray was treated with a secondary antibody to take into account background interactions with over 20,000 proteins. The microarray was then incubated with patient’s IgG (index patient 1) at a concentration of 10 μg/mL, followed by staining with goat anti-human IgG (H + L) DyLight680 (Thermo Fisher) for signal detection. The background signals from the secondary antibody were subtracted from the sample signals.

#### Cell-based assay screening

In order to determine reactivity of patients’ sample to human SYP, HeLa cells were transfected with mCer-hSYP (human SYP), kindly provided by Prof. Mike Cousin (University of Edinburgh),^59^ rSYP-YFP (rattus SYP), kindly supplied by Dr. Hisashi Umemori (Harvard Medical School) or CD2-EmGFP (Lenti ORF clone of Human CD2 molecule, Origene), separately, using lipofectamine 2000 (Thermo Fisher) according to manufacturer’s instructuion. The HeLa cells were permeabilized with PBS containing 0.2% Saponin for 15 min at room temperature after fixation with 4% PFA for 10 min at room temperature and washing with PBS. The cells were blocked with permeabilization buffer with 2% BSA for 1 h at room temperature. Subsequently, patients’ sera were constituted in permeabilization buffer at a 1:50 dilution with 0.01% BSA and applied to the cells for 2 h at room temperature. Following three washes with PBS, the reactivity was detected using a goat anti-human IgG (H + L) cross-adsorbed secondary antibody, Alexa Fluor 647 (Invitrogen, 1:400). Lastly, the cells were stained with DAPI solution (Thermo Scientific) and washed with PBS three times before imaging. Cell-based assays were performed on IBIDI 96-well round polymer plates (IBIDI).

#### Motorized fluorescent microscope with AFC

The IBIDI 96-well round polymer plates were imaged with an inverted motorized fluorescent microscope with adaptive focus control (AFC). Images were acquired with a 20×/0,80 objective and recorded with Leica DFC9000 GT sCMOS camera. Samples were excited with a Spectra X from Lumencor through a Quad SpectraX filter containing 390/22 for DAPI, 470/24 for GFP and 640/30 for AF647. Eight randomly selected regions were imaged in each well. Analysis of the motorized fluorescence microscope was compared to the evaluation of two independent blinded clinical investigators (S.H and S.M) following the scoring performed by S.M in the previous studies for MOG IgG, AQP4 IgG and Caspr2 IgG.^60^^,^^61^^,^^62^^,^^63^ Samples were considered positive if they showed binding to SYP transfected cells but not control cells or non transfected cells.

#### Depletion of anti-SYP antibodies

To deplete anti-SYP antibodies from patient’s IgG, HeLa cells were transfected with mCer-hSYP plasmid using lipofectamine 2000 (Thermo Fisher). Upon repeated immersions of patient’s IgG (400 μg/mL) on the fixed and permeabilized mCer-hSYP overexpressing HeLa cells, SYP-reactive antibodies were captured by the cells and hence depleted from the IgG sample. HeLa cells were permeabilized using PBS with 0.05% Saponin. In parallel, we also incubated patient’s IgG with irrelevant-protein (CD2-emGFP) overexpressing HeLa cells to control for the inevitable sample loss throughout repeated immersion. Complete depletion was confirmed with abolishment of immunocytochemical reactivity to mCer-hSYP expressing HeLa cells as well as abolished immunohistochemical reactivity to primate cerebellar tissue and human iPSC-derived glutamatergic neurons.

#### Immunocytochemistry

To investigate whether incubation of human iPSC-derived glutamatergic neurons with patient’s IgG would impact the localization of endogenous SYP, we utilized ioGlutamatergic neurons (Cat. no. io1001, bit.bio, Cambridge, United Kingdom). bit.bio produced these cells using deterministic cell programming.^28^ The ioGlutamatergic Neurons have rostral CNS identity.

The culture was maintained as instructed by the user manual in the 24-well-plate format. On day 16, 18 and 21, the neurons were incubated with either patient’s IgG or healthy control’s IgG at 400 μg/mL for 75 min at 37°C with 5% CO2 and washed with PBS before fixation with 4% PFA at room temperature for 10 min. The level of surface SYP was determined under non-permeabilized conditions with an anti-SYP antibody (one hour at room temperature) that recognizesthe intravesicular fragment of SYP (amino acids 50–106). Following three washes with PBS, donkey-*anti*-mouse AF488 antibody (Thermo Fisher) was applied for 1 h at room temperature under non-permeabilized conditions. The antibodies were constituted in PBS with 3% BSA. After washing, anti-MAP2 (Abcam) in PBS with 3%BSA and 0.1% Triton X-100 was applied to the neurons for 1 h at room temperature under permeabilized conditions to visualize the neuronal dendrites. This was followed by washes and incubation with goat anti-chicken IgY- AF594 for 1 h at room temperature before DAPI staining in PBS for 5 min at room temperature. To detect the total expression of SYP (intracellular and extracellular), the same protocol was followed, except all antibodies were constituted in PBS with 3% BSA and 0.1% Triton X-100 to stain under permeabilized condition. Permeabilization and cross-reactivity controls were achieved by omitting the permeabilization buffer or primary antibodies individually. Lastly, the coverslips were mounted on microscopic slides with Vectashield HardSet Antifade mounting media (VectorLab). To confirm the presynaptic location of SYP displayed on the surface, hiPSC-derived glutamatergic neurons following incubation with patient’s IgG and staining of anti-SYP under non-permeabilized condition were subsequently co-stained with anti-vGLUT1 under permeabilized condition.

#### Confocal microscopy

Confocal microscopy was conducted using an inverted Leica SP8X WLL microscope. The microscope was equipped with a 405 nm laser, a WLL2 laser (470–670 nm), and an acousto-optical beam splitter. Images were captured with a 40×/1.3 objective, yielding a pixel size of 80 nm. The fluorescence settings were as follows: DAPI (excitation 405 nm; emission 410–470 nm), GFP (489 nm; 492–550 nm), Cy3 (558 nm; 560–600 nm), and Cy5 (650 nm; 652–700 nm). To prevent bleed-through, recordings were made sequentially. GFP and Cy3 were recorded using hybrid photo detectors (HyDs) while DAPI and Cy5 were captured with conventional photomultiplier tubes. Eight randomly selected regions within the neuronal culture were imaged to yield a z stack of 10 pictures with 0.347 step increments with dimensions of 387.5 μm × 387.5 μm (1024 pixels × 1024 pixels) to encompass the diameter of a typical dendrite.

#### Multi-electrode-array

In order to evaluate the effect of anti-SYP antibodies on human iPSC-derived glutamatergic neurons’ electrophysiological activities, we measured the populational neuronal activity of the neurons with a MEA before and during treatment with index patient 1’s IgG compared to healthy control’s IgG as well as mouse anti-SYP monoclonal antibody compared to an isotype monoclonal antibody control along with a vehicle control (PBS).

Neural field potential recordings were performed using the Maestro Pro MEA platform (Axion BioSystems, USA) at 37°C and 5% CO_2_. Real-time data was collected simultaneously across all electrodes at 12.5 kHz sampling rate and 200–3000 Hz band-pass to isolate action potentials (spikes).

On day 27 and 30, a baseline recording was performed from every well before incubation with either patient’s IgG at 400 μg/mL, healthy control’s IgG at 400 μg/mL, anti-SYP mIgG (Biotechne) at 20 μg/mL, isotype mIgG control (Biotechne) at 20 μg/mL or the equivalent volume of PBS as a vehicle control. During treatment, the neurons were recorded for 5 min every 30 min for four hours. With polyclonal antibodies, the experiment was performed three times with three different differentiation rounds of human iPSC-derived glutamatergic neurons. The first experiment was performed in triplicates per condition, the second experiment was performed with nine-replicates per condition and the third experiment was performed with three-replicates per condition. With monoclonal antibodies (mIgG), the experiment was performed twice with two different differentiation rounds of human iPSC-derived glutamatergic neurons. The first experiment was performed in triplicates per condition and the second experiment was performed with six-replicates per condition. At the end of each experiment, the neurons were treated with 6,7-dinitroquinoxaline-2,3-dione (DNQX), an AMPA-receptor antagonist. The abolishment of all network activities in the presence of DNQX confirmed the glutamatergic identity of the culture.

### Quantification and statistical analysis

#### Quantification of cell-based assay screening

Analyses were performed on three-channel images (DAPI for nuclei, GFP for antigen expression, and AF647 for antibody reactivity) acquired from eight regions per well using FIJI. In each image, area of antibody reactivity was defined by the presence of AF647 signal, identified through thresholding to quantify total signal. Transfected area is determined by pixels where GFP signal is over a certain threshold. Areas with DAPI signal was excluded from the transfected area. Untransfected area is defined by all pixels with AF647 signal that are not already previously defined as transfected. To evaluate the specific reactivity of each sample to the transfected protein, the reactive index is calculated by the ratio of mean gray intensity (MGI) of AF647 channel in the transfected area to the untransfected area for every region (*i*).

$${Reactive\;index\;\left(RI\right)}_{i}=\frac{{MGI\;in\;the\;SYP\;transfected\;area}_{i}}{{MGI\;in\;the\;untransfected\;area}_{i}}$$

$$Averaged\;reactive\;index=\frac{{RI}_{1}+{RI}_{2}+{RI}_{3}+{RI}_{4}+{RI}_{5}+{RI}_{6}+{RI}_{7}+{RI}_{8}\;}{8}$$

The cut-off for positivity was defined by three standard deviations above the mean averaged reactive index of 60 healthy control samples.

#### Quantification of immunocytochemistry

Quantification of intensities at MAP2+ pixels, representing presynapses proximal to dendrites was done using FIJI with the attached Macro script (Data S1). Three biological replicates were performed and eight regions per replicate were imaged and quantified. First, the pixel intensity of all 10 optical sections of the z stack were summed up, then the pixel intensity of SYP reactivity was determined for pixels with both SYP and MAP2 reactivity, indicating mature synapses proximal to dendrites. This approach parallels prior methodologies quantifying mature synapses by staining of synapsin, which is a regulator of synaptic vesicle trafficking in presynaptic terminals,^64^ along MAP2-positive dendrites.^65^ Mann Whitney test was used to determine statistical differences on levels of surface SYP between neurons incubated with the patient’s IgG and healthy control’s IgG. P-value below 0.05 was considered significant. Statistical methods and the corresponding *n* values are provided in the figure legends. Statistical significance was denoted as follows: N.S., not significant; *p* < 0.05 (∗), *p* < 0.01 (∗∗), *p* < 0.001 (∗∗∗), *p* < 0.0001 (∗∗∗∗). Graphpad Prism and Rstudio were used for statistical analyses. Statistical details are also provided in the figure legends.

#### Data analysis of MEA

Analysis was performed using Axis Navigator software and data regarding the spike, burst and network burst activities of all wells were exported to.csv files using the Neural Metric Tool 4.1.1 and analyzed further with R version 4.1.3 and SPSS version 26. For each well, percentage change from baseline was calculated for every parameter, e.g., number of spikes, using this formula:

Percent change (number of spikes) = (number of spikes during treatment – number of spikes before treatment)/number of spikes before treatment ∗100.

To account for the nested experimental design with potential batch effects (round of experiment) and repeated measurements over time, we used a generalized linear mixed model to evaluate the effects of different antibody treatments on the neurons. A gamma distribution with a log link function was employed to model the positive, skewed response variable. Clustering per experimental round was incorporated using random effects and repeated measurements within each well, incorporating time as a fixed effect. To meet the positive value requirement of the gamma distribution, a constant offset equal to the next highest integer above the smallest observed value was added to the response variable. Statistical methods and the corresponding *n* values are provided in the figure legends. Statistical significance was denoted as follows: n.s., not significant; *p* < 0.05 (∗), *p* < 0.01 (∗∗), *p* < 0.001 (∗∗∗), *p* < 0.0001 (∗∗∗∗). Data was analyzed using SPSS version 26.

## Supplemental information

Document S1. Figures S1–S10, Tables S1–S3, Methods S1 and S2, and Data S1

Document S2. Article plus supplemental information
