# Supplementary material for: Synaptophysin autoantibodies mediate synaptic dysfunction in cerebellar ataxia
Source: Cell Rep Med. 2026 May 18;7(6):102822. doi: 10.1016/j.xcrm.2026.102822 (PMC13293949; doi:10.1016/j.xcrm.2026.102822)
Supplement: Document S1. Figures S1–S10, Tables S1–S3, Methods S1 and S2, and Data S1 [file mmc1.pdf]

Cell Reports Medicine, Volume *7*

# Supplemental information

# Synaptophysin autoantibodies mediate synaptic dysfunction in cerebellar ataxia

**Samantha Ho, Hoi Kiu Wong, Dorina Shqau, Stephan Winklmeier, Marcus Grobe-Einsler, Kevin Rostasy, Tania Kümpfel, Franziska S. Thaler, Lior Brimberg, Marc A. Schneider, Thomas Muley, Atay Vural, Thomas Seifert-Held, Verena Endmayr, Romana Höftberger, Jennifer Faber, Thomas Klopstock, Silke Frahm, Lisa Ann Gerdes, Edgar Meinl, and Simone Mader**

## Document S1. Figure S1-S10. Table S1-S3. Method S1-S2.


Figure S1. Autoantibodies of index patient 1 colocalize with presynaptic markers on excitatory neurons, related to Figure 1

Figure S2. Additional regions of co-immunohistochemical staining on primate cerebellar cortex, related to Figure 1

Figure S3. Characterization of index patient 1's SYP-reactive antibodies, related to Figure 1

Figure S4. Additional regions of co-immunohistochemical staining on primate cerebellar cortex, related to Figure 1

Figure S5. Cell-based assay results of two SYP IgG positive index patients and titration of index patient 1, related to Figure 2

Figure S6. Validation of SYP-reactive IgG depletion and colocalization of patient's IgG and vGLUT2 on hiPSC-derived glutamatergic neurons, related to Figure 4

Figure S7. Brain MRI of Index Patient 1 showing cerebellar atrophy, related to Figure 3

Figure S8. Immunocytochemistry controls, related to Figure 5

Figure S9. Permeabilized SYP staining of hiPSC-derived glutamatergic neurons after incubation, related to Figure 5

Figure S10. Raster plot from MEA recordings of hiPSC-derived glutamatergic neurons, related to Figure 6

Table S1. Top 40 reactive proteins to index patient 1's purified IgG using HuProt v4.0 - Proteome array

Table S2. Clinical cohorts screened for reactivity to SYP using cell-based assay

Table S3. Detailed information on the two index patients

Method S1. Distinct clinical features of two index patients with SYP autoantibodies, related to Table S3.

Method S2. Disclosures

Data S1. ImageJ Macroscript for quantification, related to Figure 5.

A

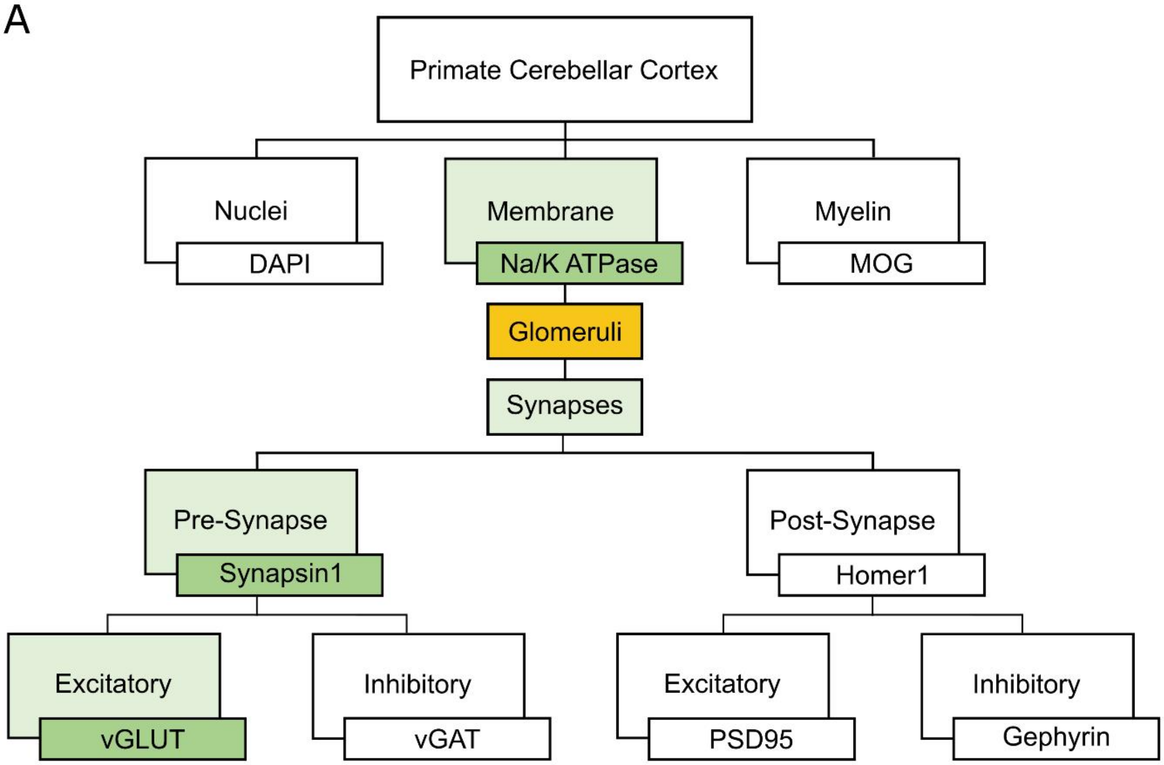
Primate Cerebellar Cortex
Nuclei
DAPI
Membrane
Na/K ATPase
Myelin
MOG
Glomeruli
Synapses
Pre-Synapse
Synapsin1
Post-Synapse
Homer1
Excitatory
vGLUT
Inhibitory
vGAT
Excitatory
PSD95
Inhibitory
Gephyrin

B

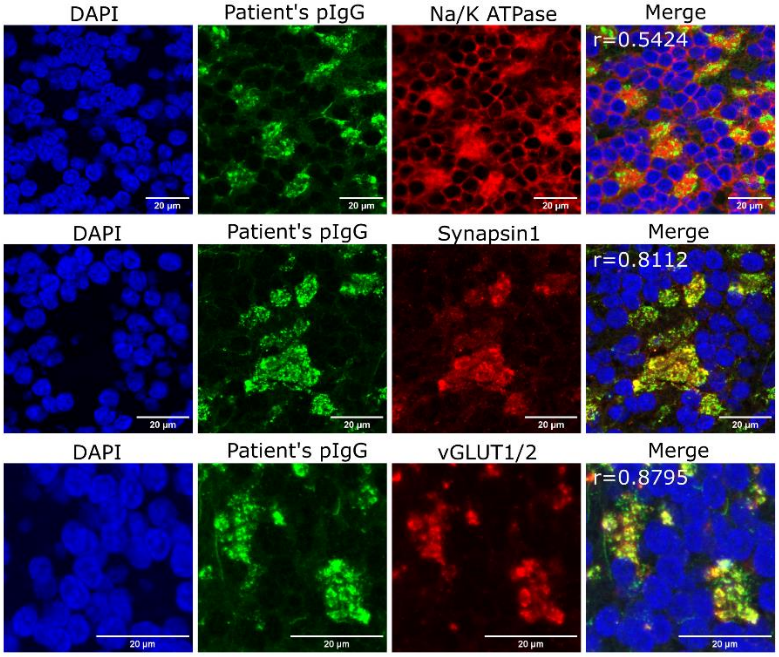
DAPI
Patient's pIgG
Na/K ATPase
Merge
r=0.5424
DAPI
Patient's pIgG
Synapsin1
Merge
r=0.8112
DAPI
Patient's pIgG
vGLUT1/2
Merge
r=0.8795
20 µm

**Figure S1. Autoantibodies of index patient 1 colocalize with presynaptic markers on excitatory neurons, related to Figure 1**

(A) Strategy to localize cell type expression of autoantigen using co-immunohistochemistry. (B) Co-localization of patient's purified IgG (green, 400µg/ml) with membrane marker (Na/K ATPase; red; top), presynaptic marker (Synapsin1; red; middle) and excitatory presynaptic marker (vGLUT1/2; red; bottom) alongside DAPI (blue). Colocalization between green and red images were quantified by Pearson's coefficient (*r*), calculated with ImageJ Colocalization Finger plug-in ($r = 1$: complete colocalization; $r = 0$: absence of colocalization). Scale bars represent 20µm. Figure created with Inkscape.

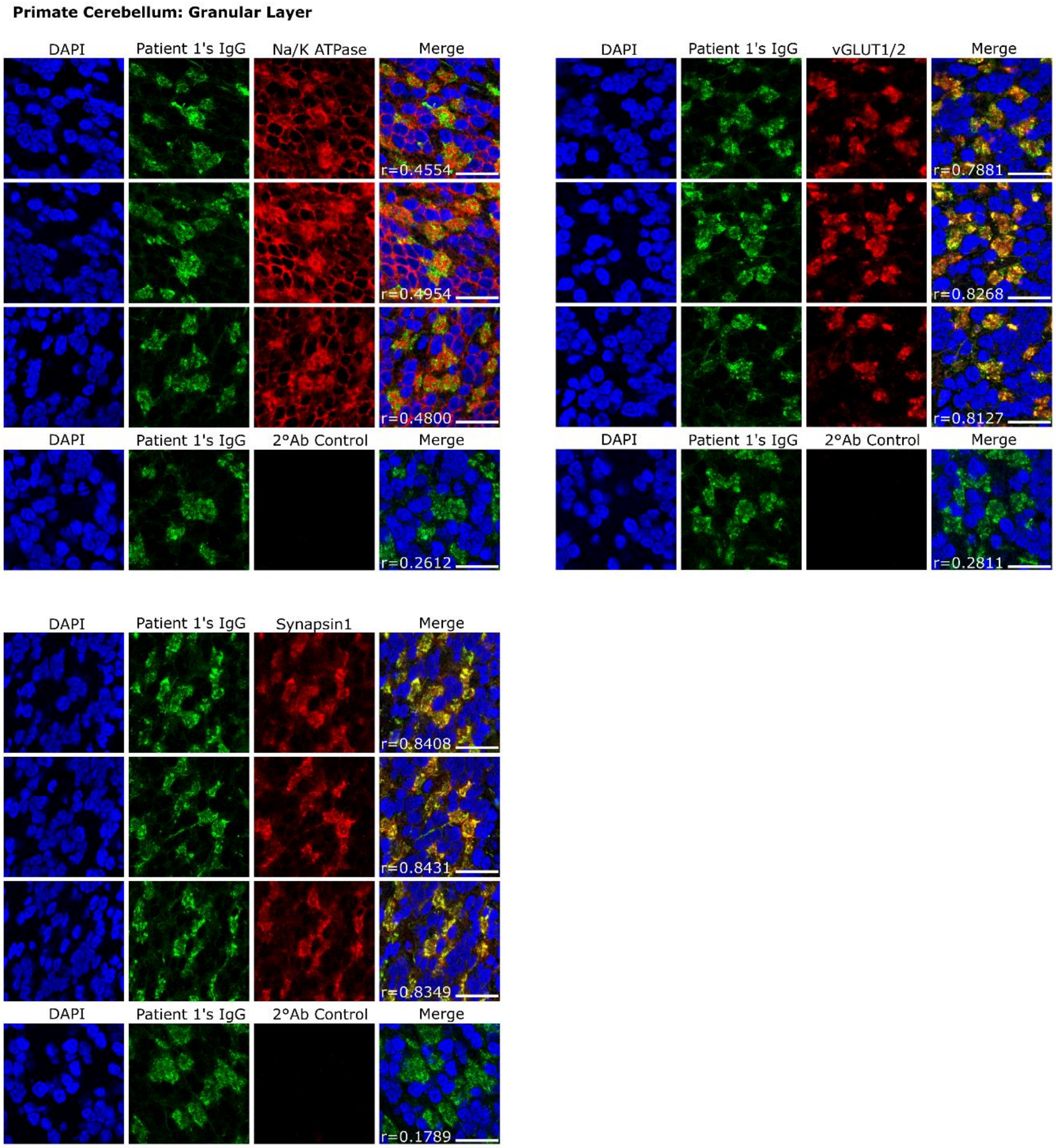


**Figure S2. Additional regions of co-immunohistochemical staining on primate cerebellar cortex, related to Figure 1**

Co-immunohistochemistry of index patient 1's purified IgG (green; 400µg/ml) with commercial anti-Na/K ATPase (red; top left), anti-Synapsin1 (red; bottom left) and anti-vGLUT1/2 (red; top right) antibodies and DAPI (blue) on primate cerebellar tissue. Scale bars indicate length of 20µm. Colocalization between green

and red images were quantified by Pearson's coefficient (*r*), calculated with ImageJ Colocalization Finger plug-in (*r* = 1: complete colocalization; *r* = 0: absence of colocalization). Figure created with Inkscape.

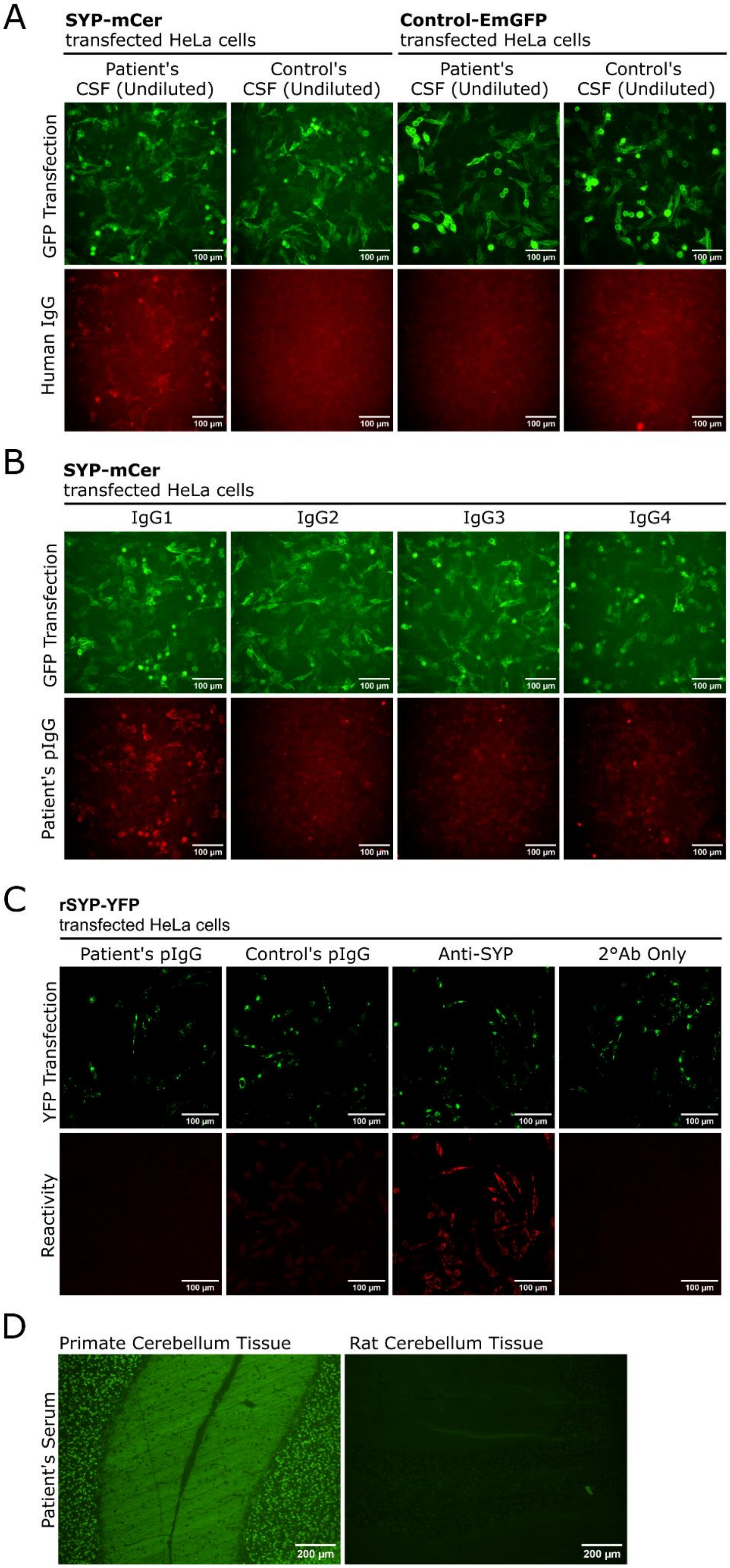
A
SYP-mCer
transfected HeLa cells
Control-EmGFP
transfected HeLa cells
Patient's CSF (Undiluted)
Control's CSF (Undiluted)
Patient's CSF (Undiluted)
Control's CSF (Undiluted)
GFP Transfection
Human IgG
100 µm
B
SYP-mCer
transfected HeLa cells
IgG1
IgG2
IgG3
IgG4
GFP Transfection
Patient's pIgG
C
rSYP-YFP
transfected HeLa cells
Patient's pIgG
Control's pIgG
Anti-SYP
2°Ab Only
YFP Transfection
Reactivity
D
Primate Cerebellum Tissue
Rat Cerebellum Tissue
Patient's Serum
200 µm

**Figure S3. Characterization of index patient 1's SYP-reactive antibodies, related to Figure 1**

(A) Immunocytochemistry of index patient 1's and healthy control's undiluted cerebrospinal fluid (CSF; red) on SYP-mCer (antigen)- and CD2-EmGFP (control protein)- transfected HeLa cells (green). Scale bars indicate 100µm. (B) Detection of reactive IgG subclass in index patient 1's purified IgG to SYP-mCer transfected HeLa cells (green) using secondary antibodies selective to human IgG1, IgG2, IgG3 and IgG4 (red). Scale bars indicate 100µm. (C) Immunocytochemistry of commercial anti-SYP antibody, index patient 1's and healthy control's purified IgG (400µg/ml; red) in rat SYP-YFP transfected HeLa cells (green). Scale bars indicate 100µm. (D) Immunohistochemistry of index patient 1's serum (1:20) to primate and rat cerebellar tissue sections. Scale bars indicate 200µm.Figure created with Inkscape.

**Primate Cerebellum: Granular Layer**

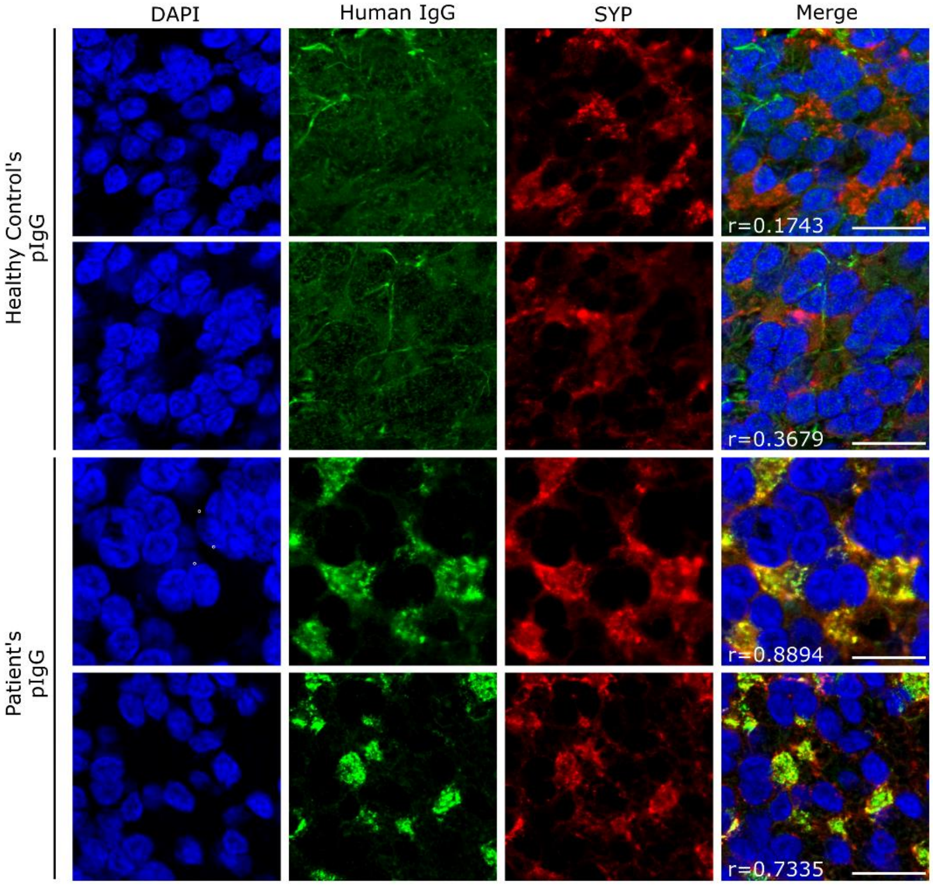


**Primate Cerebellum: Purkinje Cell Body**

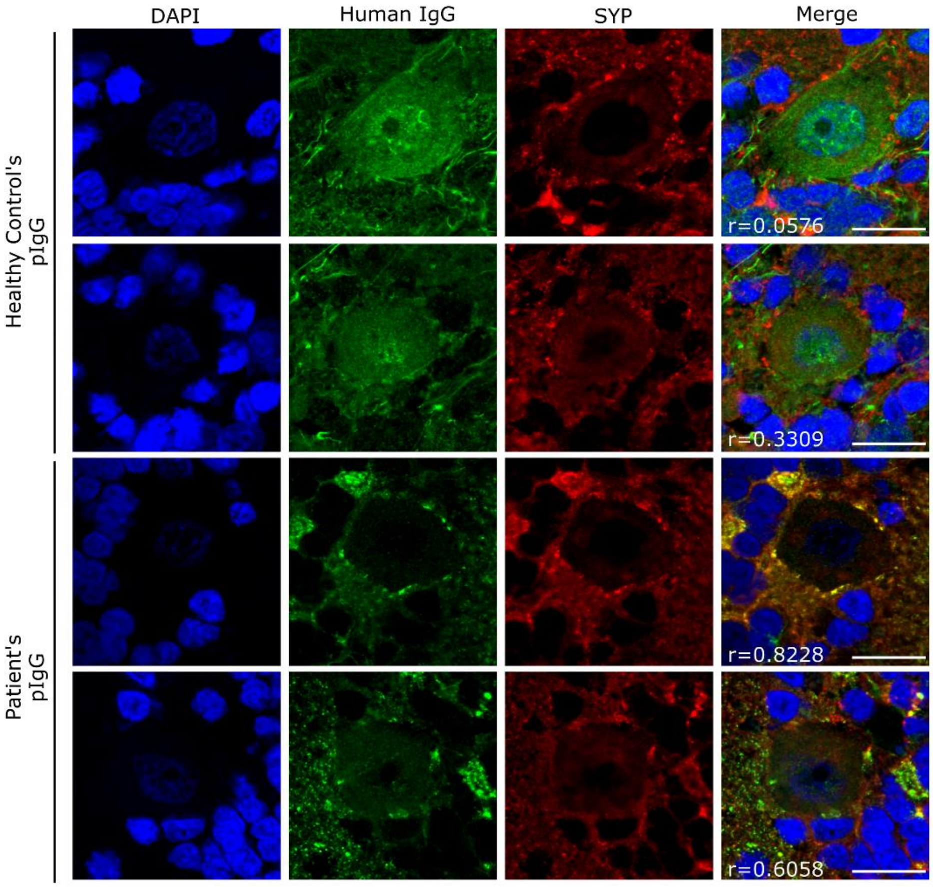

**Figure S4. Additional regions of co-immunohistochemical staining on primate cerebellar cortex, related to Figure 1**

Co-immunohistochemistry of healthy control's and index patient 1's purified IgG (green; 400µg/ml) with commercial anti-SYP monoclonal antibodies (red; 1:100; Bio Techne) and DAPI (blue) on primate cerebellar tissue. Scale bars indicate length of 20µm. Colocalization between green and red images were quantified by Pearson's coefficient (*r*), calculated with ImageJ Colocalization Finger plug-in (*r* = 1: complete colocalization; *r* = 0: absence of colocalization). Figure created with Inkscape.

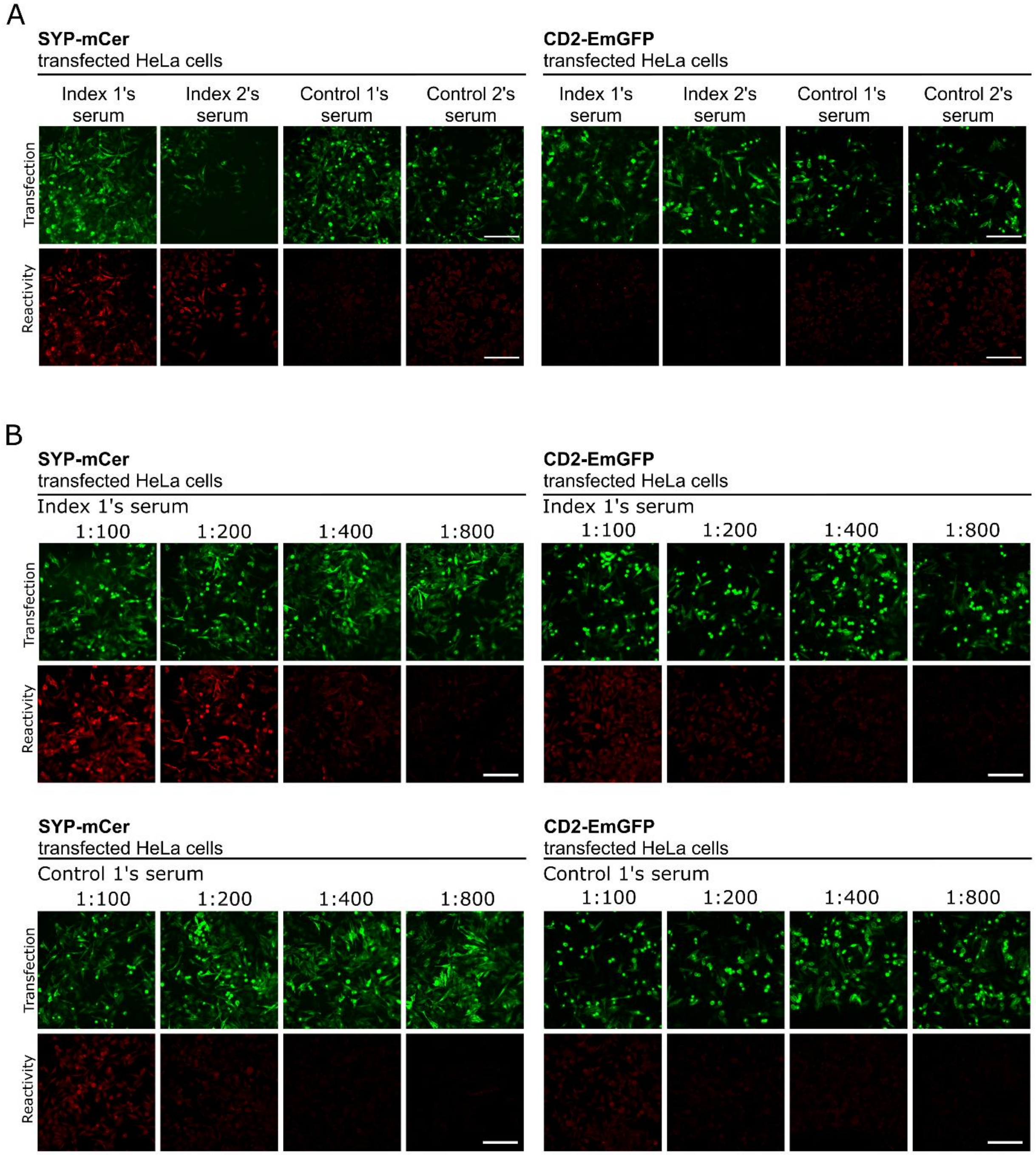


**Figure S5. Cell-based assay results of two SYP IgG positive index patients and titration of index patient 1, related to Figure 2**

(A) Immunocytochemistry of index patient 1's, index patient 2's, and two healthy control's serum (1:20) on human SYP-mCer (antigen)- and human CD2-EmGFP (control protein)- transfected Hela cells. Healthy control 1 exemplifies a serum sample that displayed low unspecific reactivity to transfected HeLa cells whereas healthy control 2 exemplifies a serum sample that exhibited high level of unspecific IgG reactivity. Scale bars indicate length of 200µm. (B) Immunocytochemistry of serum from index patient 1 and one healthy control on human SYP-mCer (antigen)- and human CD2-EmGFP (control protein)- transfected Hela cells with titrations. Scale bars indicate length of 200µm. Figure created with Inkscape.

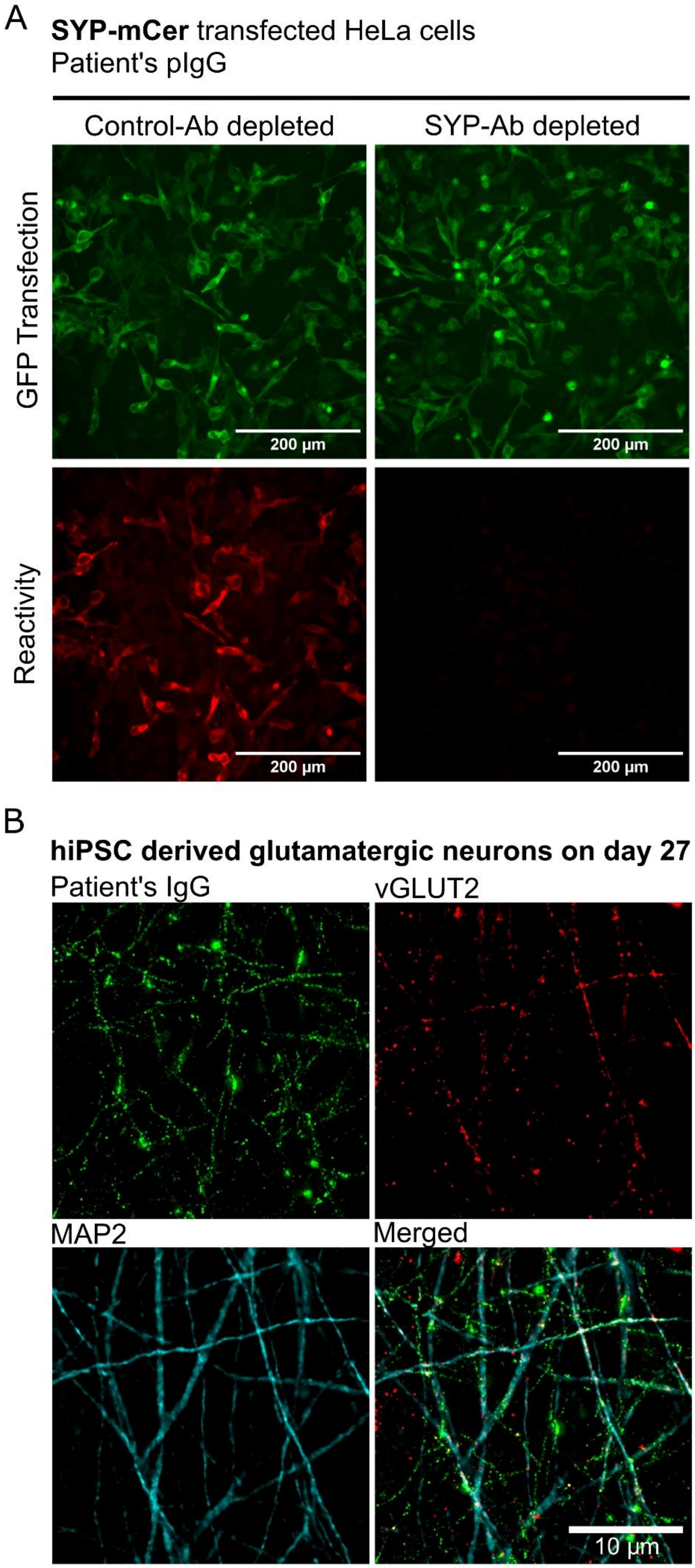
A
SYP-mCer transfected HeLa cells
Patient's pIgG
Control-Ab depleted
SYP-Ab depleted
GFP Transfection
Reactivity
200 µm
200 µm
200 µm
200 µm
B
hiPSC derived glutamatergic neurons on day 27
Patient's IgG
vGLUT2
MAP2
Merged
10 µm

**Figure S6. Validation of SYP-reactive IgG depletion and colocalization of patient's IgG and vGLUT2 on hiPSC-derived glutamatergic neurons, related to Figure 4**

(A) Immunocytochemistry of Control-Ab-depleted and SYP-Ab-depleted fractions of the patient's purified IgG. Depletion was performed by preabsorbing SYP-reactive antibodies by incubating with SYP-mCer transfected HeLa cells. Control depletion was performed by incubating patient's pIgG with CD2-EmGFP transfected HeLa cells. Scale bars indicate length of 200µm. (B) Immunocytochemical detection of extracellular reactivity by Index Patient 1's IgG (400µg/ml; green), intracellular vGLUT2 (red) and intracellular MAP2 (cyan) in hiPSC-derived glutamatergic neurons on day 27. Scale bars indicate length of 10µm. Figure created with Las X and Inkscape.

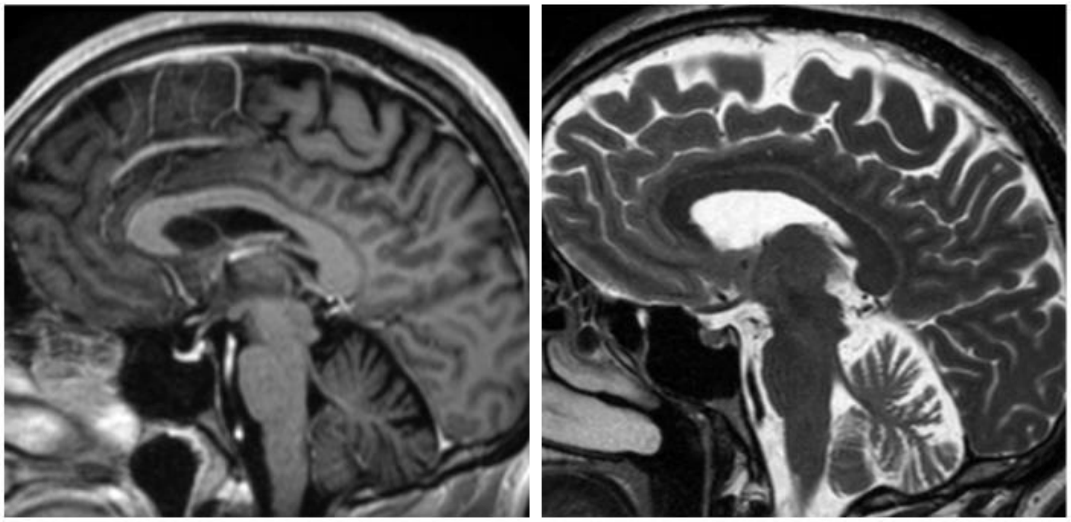

**Figure S7. Brain MRI of Index Patient 1 showing cerebellar atrophy, related to Figure 3**

Left: Axial T1-weighted image demonstrating cerebellar atrophy.

Right: Sagittal T2-weighted image highlighting cerebellar atrophy.

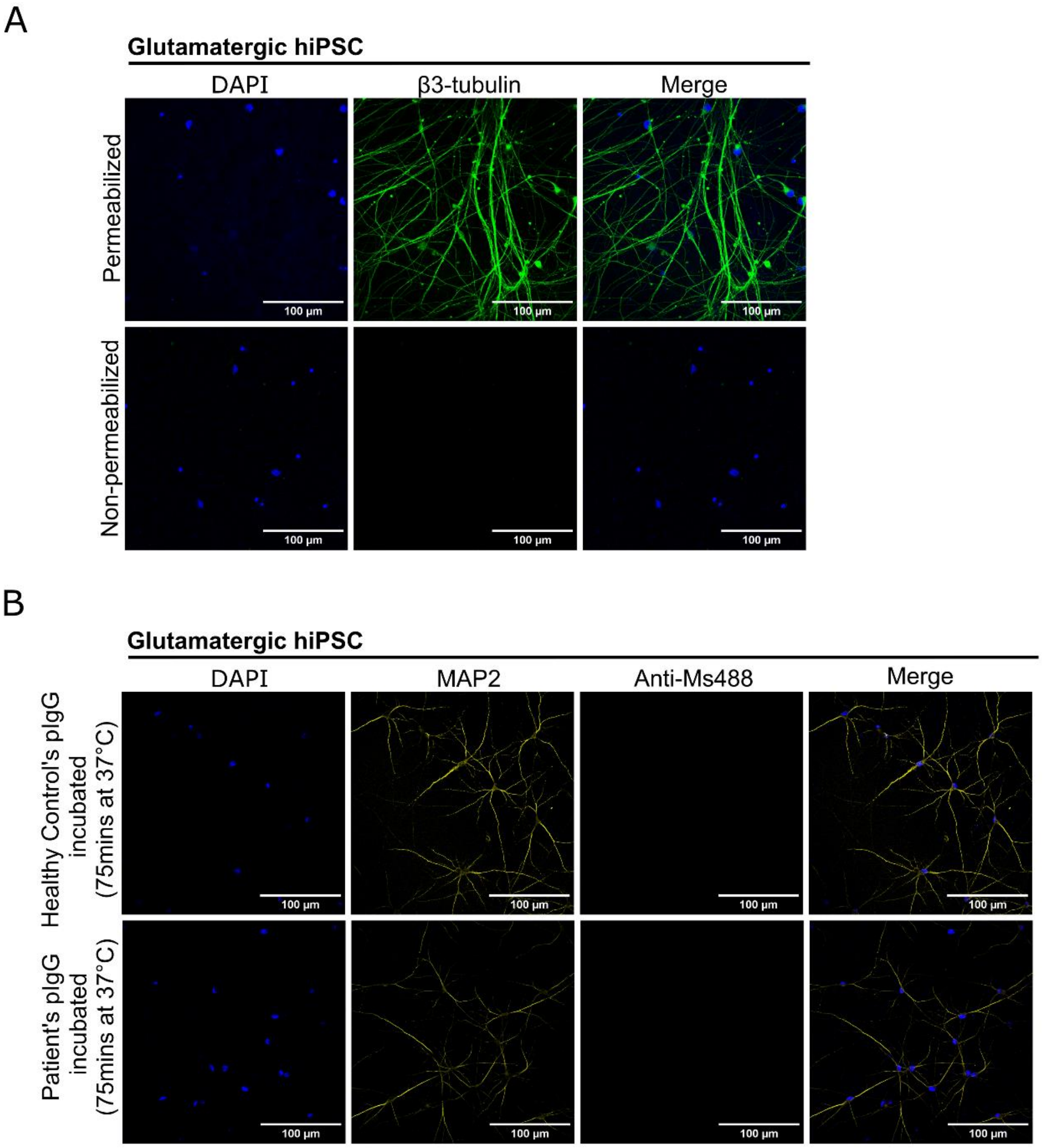


**Figure S8. Immunocytochemistry controls, related to Figure 5**

(A) Immunocytochemical staining of intracellular marker, beta-3-tubulin (green) alongside DAPI (blue) on hiPSC-derived glutamatergic neurons under permeabilized and non-permeabilized condition to confirm that our non-permeabilized protocol preserved the membrane integrity. Scale bars indicate length of 100µm. (B) To ensure that the anti-mouse AF488 secondary antibody did not cross-react with patient's or healthy control's pIgG, anti-mouse AF488 antibody (green) was stained in the absence of anti-SYP antibodies in

the neurons following incubation without permeabilization. Subsequently, anti-MAP2 (yellow) and DAPI (blue) were applied to visualize the neuron after permeabilization. Scale bars indicate length of 100μm. Figure created with Inkscape.

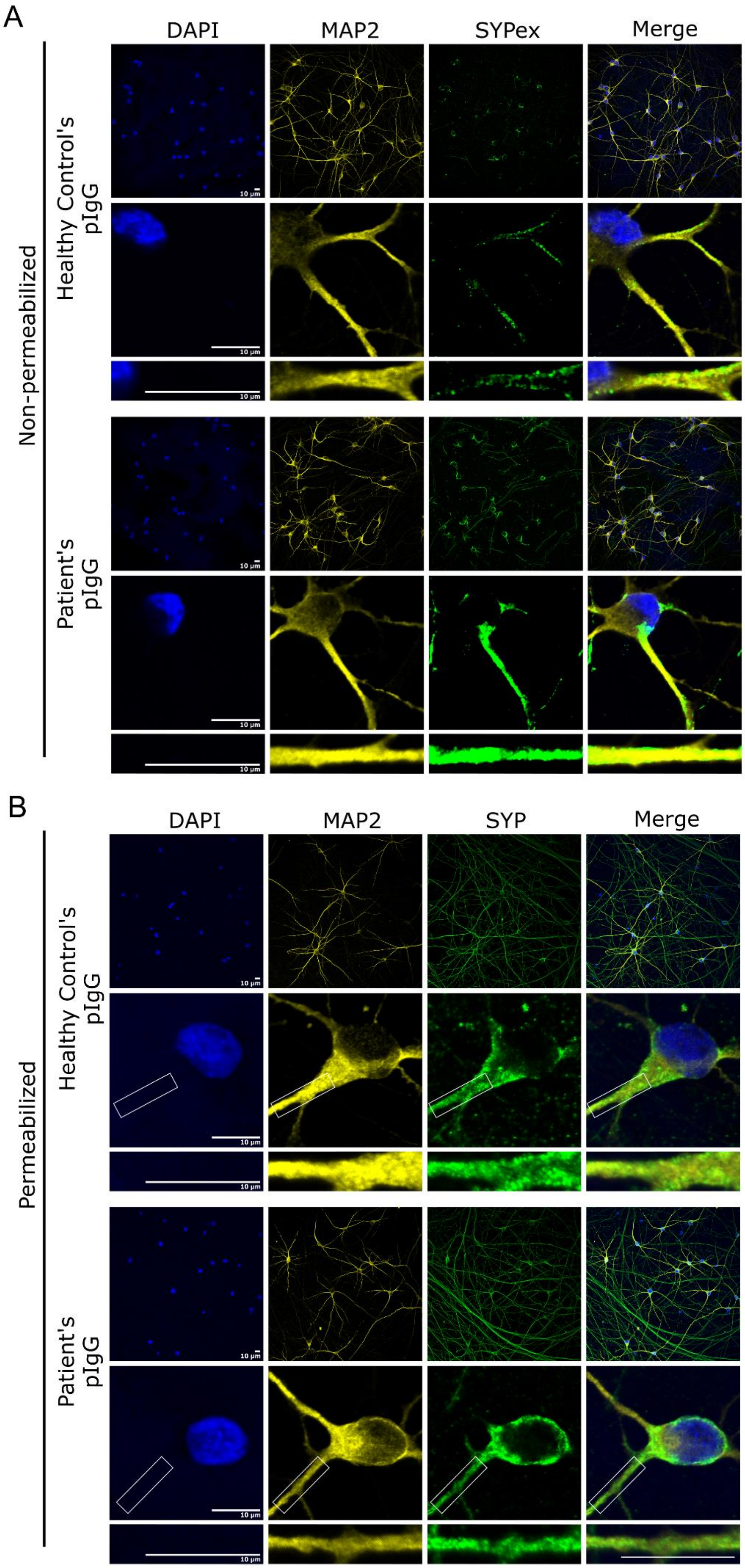
A
DAPI
MAP2
SYPex
Merge
Non-permeabilized
Healthy Control's pIgG
Patient's pIgG
10 µm
B
DAPI
MAP2
SYP
Merge
Permeabilized
Healthy Control's pIgG
Patient's pIgG
10 µm

**Figure S9. Permeabilized SYP staining of hiPSC-derived glutamatergic neurons after incubation, related to Figure 5**

Immunocytochemical detection of extracellular SYP (a; green) and total level of SYP (b; green) in hiPSC-derived glutamatergic neurons following incubation with healthy control's or index patient 1´s purified IgG (400µg/ml) under non-permeabilized (A) and permeabilized (B) conditions alongside MAP2 (yellow) and DAPI (blue). For each condition, a higher-magnification image and a zoomed-in view of the boxed region (bottom row) are shown with their indicated scale bars, representing 10µm. Figure created with Inkscape.

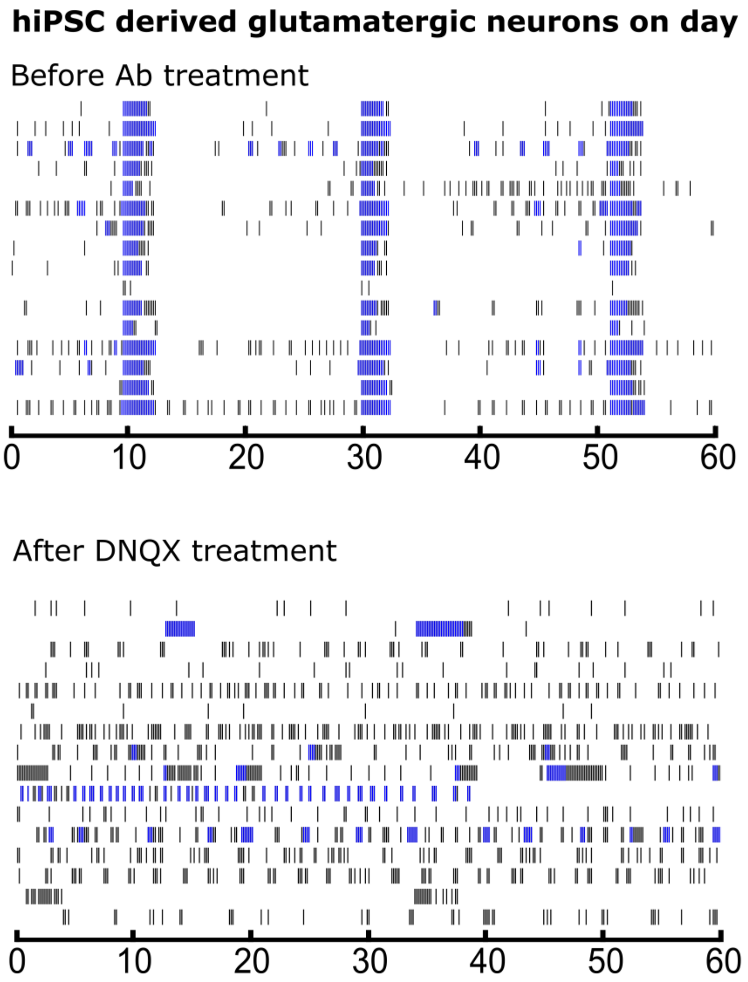


**Figure S10. Raster plot from MEA recordings of hiPSC-derived glutamatergic neurons, related to Figure 6**

Representative 60-seconds raster plots from the MEA recordings of hiPSC-derived glutamatergic neurons on the day of experiment (day 27 and day 30) before treatment application and after application of a glutamate receptor antagonist, DNQX. The unit of the x-axis is seconds. Spikes are represented with black lines and bursts are represented with blue lines. Figure created with Neural Metric Tool (Axion) and Inkscape.

**Table S1. Top 40 reactive proteins to index patient 1's purified IgG using HuProt v4.0 - Proteome array**

| Rank | Name | UniProt ID | Intensity values |
|---|---|---|---|
| 1 | UBQLN2 | Q9UHD9 | 5.161,0 |
| 2 | FAM29A | | 3.793,5 |
| 3 | NCOA3 | Q9Y6Q9 | 2.953,0 |
| 4 | ANP32A | P39687 | 2.631,0 |
| 5 | RAP1GAP | P47736 | 2.552,0 |
| 6 | PRRC2B | Q5JSZ5-2 | 2.515,0 |
| 7 | UBQLN3 | Q9H347 | 2.488,0 |
| 8 | CCDC102B_frag | | 2.445,0 |
| 9 | GTSF1L | Q9H1H1-2 | 2.380,0 |
| 10 | LRRFIP1 | Q32MZ4 | 2.369,0 |
| 11 | NG_015859.1_frag | | 2.271,0 |
| 12 | ANKRD45 | Q5TZF3-2 | 2.229,0 |
| 13 | ZSCAN5A | Q9BUG6 | 2.136,0 |
| 14 | IRAK4 | Q9NWZ3 | 2.128,0 |
| 15 | UBL7 | Q96S82 | 2.042,0 |
| 16 | ZSCAN5A | Q9BUG6 | 2.008,0 |
| 17 | OMP | P47874 | 1.890,0 |
| 18 | RAP1GAP | | 1.870,0 |
| 19 | PAFAH1B2 | P68402 | 1.796,0 |
| 20 | Hep B Protein X | | 1.745,0 |

| | | | |
|---|---|---|---|
| 21 | SLC30A9 | Q6PML9 | 1.547,0 |
| 22 | TRGC1 | P0CF51 | 1.517,0 |
| 23 | BC073758 | | 1.399,0 |
| 24 | FSCN1 | Q16658 | 1.307,0 |
| 25 | BC054893.1_frag | | 1.267,0 |
| 26 | SH3GL1 | Q99961 | 1.218,0 |
| 27 | IGLL5 | | 1.169,0 |
| 28 | CT47A1 | Q5JQC4 | 1.075,0 |
| 29 | ATCAY | Q86WG3 | 1.057,0 |
| 30 | MPST | P25325 | 1.048,0 |
| 31 | PPP1R12B | O60237-2 | 1.038,0 |
| 32 | PCSK7 | | 991,0 |
| 33 | KJ905804 | | 899,0 |
| 34 | SYP | P08247 | 885,0 |
| 35 | IGL@ | | 842,0 |
| 36 | BRD9 | Q9H8M2-2 | 841,0 |
| 37 | TMEM106B | Q9NUM4 | 823,0 |
| 38 | TACC1 | O75410-7 | 811,0 |
| 39 | NRF1 | Q16656-4 | 805,0 |
| 40 | VRK2 | Q86Y07 | 791,0 |

List of 40 most reactive protein candidates to index patient's purified IgG (400µg/ml) in the HuProt Human Proteome microarray v4.0 protein array. The intensity values were determined by the extent of anti-human IgG antibody in the microarray normalized to unspecific interaction with the secondary antibody. SYP appeared as candidate number 34 in the proteome array.

**Table S2. Clinical cohorts screened for reactivity to SYP using cell-based assay**

| Cohorts tested with CBA: | Serum (n=) | Age at sampling | Male/Female counts |
|---|---|---|---|
| **Healthy controls**<br>- LMU Klinikum | 60 | 32 (18-59) | 21/39 |
| **Sporadic cerebellar ataxia (no synaptic reactivity on tissue)**<br>- DZNE, LMU Klinikum, Children's Hospital of Datteln<br>* | 100 | 56 (2-76) | 61/39 |
| **Sporadic cerebellar ataxia (with synaptic reactivity on tissue)**<br>- DZNE, LMU Klinikum, Medical University of Vienna/Austria, Children's Hospital of Datteln, Mayo Clinic | 43 | 61 (6-84) | 22/21 |
| **Immune-mediated CA**<br>LMU Klinikum, DZNE | 15 | 64 (53-71) | 1/14 |
| **Other inflammatory neurological diseases with CA symptoms**<br>- LMU Klinikum, | 4 | 38 (32-40) | 2/2 |

| | | | |
|---|---|---|---|
| **Other inflammatory neurological diseases**<br><br>- LMU Klinikum, Koç University Research Center for Translational Medicine (KUTTAM), University of Vienna/Austria | 28 | 44 (18-67) | 11/17 |
| **SYP+ tumour**<br><br>- LMU Klinikum, Lung Biobank Heidelberg | 195 | 64 (41-84) | 117/78 |

Clinical cohorts of individuals whose sera were tested for IgG reactivity against SYP-mCer transfected HeLa cells using cell-based assays. Clinical samples were obtained from centers including the medical faculty of Ludwig-Maximilians-University (LMU), German Center for Neurodegenerative Diseases (DZNE), Medical University of Vienna/Austria, the Children's Hospital of Datteln and the Lung Biobank Heidelberg

* one CSF sample exhibited synaptic reactivity on tissue although the serum did not.

## Table S3. Detailed information on the two index patients

| Patient | Gender | Age | Diagnosis | Cancer | Detectable Anti-SYP IgG+ | Coexisting autoimmune disease | Brain MRI | Immunosuppressive treatment and response | Disease duration * | CSF findings | Time to Immune-suppressive treatment |
|---|---|---|---|---|---|---|---|---|---|---|---|
| Index 1 | M | 48 | Cerebellar ataxia. Progressive | None | Serum, CSF | Psoriasis | Atrophy in the cerebellar vermis | High dose steroids, plasma exchange and B-cell depletion with rituximab showed no efficacy | 1 year | -No OCB<br>-No CSF pleocytosis<br>-Elevated protein in CSF | 1 year |
| Index 2 | M | 61 | Cerebellar ataxia. Mild cognitive impairment | No tumour screening performed | Serum | None | Normal | No | >2 years | -Normal cell count<br>-Elevated total protein | No therapy |

***from first manifestation till diagnosis**

## Method S1. Distinct clinical features of two index patients with SYP autoantibodies, related to Table S3.

**Index patient 1**: A previously healthy 48 years old patient developed without any prodrome or infectious trigger a progressive cerebellar syndrome within 12 months characterized by initial gait ataxia and was followed by limb ataxia, dysarthria, and cerebellar oculomotor dysfunction that slowly progressed over the next year. Medical history was unremarkable despite psoriasis. Comprehensive evaluation ruled out both acquired and genetic causes of ataxia including antibody screening. The serum was tested negative for antibodies against AQP4, MOG, NMDA-receptor, CASPR2, Hu, Ri, ANNA-3, Yo, Tr/DNER, Myelin, Ma/Ta, GAD56, Amphiphysin, AMPA-receptor, GABA-b-receptor, LGI-1, ZIC4, DPPX, CARPVIII, Glycin-receptor, mGluR1, mGluR5, GABA-a-receptor, Rho GTPase activating protein 26, ITPR1, Homer-3, Recoverin, Neurochondrin, GluRD2, Flotillin-1/2, and IgLON5. Whole body FDG-PET did not show an underlying tumour etiology, however FDG-PET of the brain revealed a slight reduction of glucose metabolism in the right posterior putamen and a strong reduction of the cerebellar glucose metabolism (more pronounced on the left side), indicating neuronal dysfunction (Figure 3). Brain MRI showed slight cerebellar atrophy (pronounced within the midline vermis) without any other pathology (Figure S7). CSF analysis yielded elevated protein (85 mg/dl, Ref <45), a moderate disruption of the blood-brain-barrier, increased interleukin-6 and elevated neurofilament heavy chains (ELISA, 1267 pg/ml) indicating neuroaxonal damage. Additional CSF investigations yielded unremarkable results (including negative intrathecal IgG synthesis, negative oligoclonal bands and antibody index to measles, rubella, zoster (MRZ-reaction), Beta-Amyloid (1-42 and 1-40), Beta-Amyloid-Ration, Tau-protein and Phospho-Tau). Autoimmune cerebellar inflammation was suspected, however treatment with high dose steroids, plasma exchange and B-cell depletion with rituximab showed no efficacy. The patient died from severe Covid-19 infection despite intensive care including invasive ventilation 3 years after disease onset.

**Index patient 2:** Index patient 2 (male) presented at the age of 61 years with a 2 years history of gait ataxia. Further medical history was unremarkable. Clinical examination revealed oculomotor dysfunction, axial rigidity, bradykinesia, dysmetria in heel-to-shin test on both sides and ataxic gait disturbance. Reflexes were exaggerated, Babinski's sign was negative. Urinary retention required constant catheterization. Mild cognitive impairment was found on mini mental state examination (27/30). MRI of the brain showed unrelated incidental findings (right frontal post-traumatic glial scar, right cerebellar lacune). $^{123}$I-ioflupane SPECT images yielded decreased striatal uptake bilaterally. FDG-PET scan of the brain and MRI of the spinal cord were unremarkable. CSF analysis revealed normal cell count and elevated total protein 154 mg/dl (<60 mg/dl). Beta-Amyloid (1-42 and 1-40), Beta-Amyloid-Ratio, Tau-protein and Phospho-Tau in CSF were all normal. In serum and CSF, no antibodies were detected against NMDAR, AMPAR, GABA-b-receptor, LGI1, CASPR2, DPPX, IgLON5, GAD65, AK5, Homer3, PKCgamma, CARPVIII, ARHGAP26, SOX1. No anti-Hu-, anti-Yo-, anti-Ri-, anti-DNER/Tr-, anti-CV-, anti-Ma1/2- or anti-amphiphysin-antibodies were found. The patient had a degree of disability of 2 on the modified Rankin Scale (mRS). Clinical symptoms were unresponsive to levodopa. Immunosuppressive treatments were not applied. The patient was lost for follow-up.

## Method S2. Disclosures

Samantha Ho was awarded a scholarship from the Studienstiftung des deutschen Volkes. Edgar Meinl has received honoraria from Merck, Novartis, Roche, and TEVA, as well as grant support from Novartis, Roche, GlycoEra, Alexion, and Horizon, all outside of the submitted work. Simone Mader has received honoraria from Alexion and UCB, along with grant support from Alexion and Novartis, outside the scope of the submitted work. Stephan Winklmeier received funding from the Medical & Clinician Scientist Program (MCSP), the Junior Researcher Fund of LMU Munich, and the SPIN Award from Grifols S.A. These funding sources were unrelated to the present study. Atay Vural, Lior Brimberg, Hoi Kiu Wong and Dorina Shqau, Silke Frahm, Thomas Seifert-Held have no disclosures. Tania Kümpfel has received speaker fees and/or personal compensation for advisory board participation from Alexion/AstraZeneca, UCB, Merck, and Biogen, as well as for lectures and educational events from Alexion/AstraZeneca, Novartis Pharma, Roche Pharma, Horizon Therapeutics/Amgen, and Chugai Pharma. Her institution has also been compensated for its role in a steering committee with Roche. TK serves as a principal investigator for several randomized clinical trials (Novartis Pharma, Roche Pharma, BMS, and Sanofi Genzyme) and a trial funded by the BMBF (funding code: 01GM1908E). Her institution has received compensation for these clinical trials, all of which are outside the scope of this work. Franziska Thaler received grant support from Novartis and speaker honorarium from Alexion outside of the submitted work. Kevin Rostasy received consultancy fees for the Roch Operetta 2 study and honoraria for lectures from Merck. R. Höftberger reports speaker honoraria from UCB, MBS, and Euroimmun. The Medical University of Vienna (Austria; employer of Dr. Höftberger) receives payment for antibody assays and for antibody validation experiments organized by Euroimmun (Lübeck, Germany). Verena Endmayr reports no relevant disclosures. LAG has received personal fees for advisory boards from Roche Pharma, Amgen and for speaker honoraria and/or lectures/education from Alexion/Astra Zeneca, Bayer Vital,

Novartis, Merck. LAG has received a research grant from Merck. LAG is a site Co-principal investigator in several randomized clinical trials (Novartis Pharma, Roche Pharma, BMS and Sanofi Genzyme) and her institution has received compensation for clinical trials all outside the present work. MGE received research support from the German Ministry of Education and Research (BMBF) within the European Joint Program for Rare Diseases (EJP-RD) 2021 Transnational Call for Rare Disease Research Projects (funding number 01GM2110), from the National Ataxia Foundation (NAF), and from Ataxia UK, and received consulting fees from Healthcare Manufaktur, Germany, and Biogen, all unrelated to this study. Marc A. Schneider and Thomas Muley received institutional funding from ROCHE, Cellzome and Oncohost. The Lung Biobank Heidelberg is partly funded by The German Center for Lung Research (DZL, funding code 82DZL00402).

Thomas Klopstock has received honoraria from Biogen and Chiesi, as well as grant support from Khondrion, Stealth Biotherapeutics, and OMEICOS Therapeutics, all outside of the submitted work. Jennifer Faber was funded within the Advanced Clinician Scientist Programme (ACCENT, funding code 01EO2107, by the German Federal Ministry of Education and Research (BMBF) and as a PI of the iBehave Network, sponsored by the Ministry of Culture and Science of the State of North Rhine-Westphalia and received consultancy honoraria from Vico therapeutics and Biogen, unrelated to the present manuscript.

## Data S1. ImageJ Macroscript for quantification, related to Figure 5.

```
run("Set Measurements...", "area mean min stack display redirect=None decimal=3");
run("Set Scale...", "distance=2.6426 known=1 unit=µm");

imageName=getTitle();
print(imageName);

//sum all images in the stack of 3.12µm
run("Z Project...", "projection=[Sum Slices]");
run("Split Channels");

//rename each channel and turn images 8-bit
// DAPI channel: pixel value unchanged
selectWindow("C1-SUM_"+imageName);
rename("Blue");
setOption("ScaleConversions", true);
run("8-bit");
//SYPex channel: pixel value unchanged
selectWindow("C2-SUM_"+imageName);
rename("Green");
setOption("ScaleConversions", true);
run("8-bit");
setMinAndMax(0, 255);
run("Apply LUT");
//MAP2 channel, turn image binary (each pixel is either 0 or 255)
selectWindow("C3-SUM_"+imageName);
rename("Yellow");
setOption("BlackBackground", true);
run("Convert to Mask");
setOption("ScaleConversions", true);
```

```
run("8-bit");

//Measure SYPex pixels that are also MAP2+
imageCalculator("AND create", "Green", "Yellow");
run("Measure");
selectImage("Result of Green");

//Cell count using DAPI (cell size 50-1200µm2; diameter of 8µm to 40µm)
selectImage("Blue");
run("Subtract Background...", "rolling=50 sliding");
//run("Threshold...");
setThreshold(20, 255, "raw");
setOption("BlackBackground", true);
run("Convert to Mask");
run("Watershed");
run("Analyze Particles...", "size=50-1200 show=Outlines summarize");
close("*");
```
